# Frequency, characteristics and risk assessment of pulmonary arterial hypertension with a left heart disease phenotype

**DOI:** 10.1007/s00392-024-02448-9

**Published:** 2024-04-15

**Authors:** Matteo Toma, Giulio Savonitto, Carlo Maria Lombardi, Edoardo Airò, Mauro Driussi, Piero Gentile, Luke Howard, Martina Moschella, Emma Di Poi, Matteo Pagnesi, Simonetta Monti, Valentino Collini, Luciana D’Angelo, Veronica Vecchiato, Alberto Giannoni, Marianna Adamo, Davide Barbisan, Carolina Bauleo, Andrea Garascia, Marco Metra, Gianfranco Sinagra, Francesco Lo Giudice, Davide Stolfo, Pietro Ameri

**Affiliations:** 1 Cardiovascular Disease Unit, IRCCS Ospedale Policlinico San Martino, IRCCS Italian Cardiovascular Network, Largo Rosanna Benzi 10, 16132 Genoa, Italy; 2 Cardiothoracovascular Department, Azienda Sanitaria Universitaria Giuliano Isontina (ASUGI) and University Hospital of Trieste, Via Valdoni 7, 34149 Trieste, Italy; 3 Department of Medical and Surgical Specialties, Radiological Sciences, and Public Health, Cardiology, ASST Spedali Civili, University of Brescia, Brescia, Italy; 4 Cardiology and Pneumology Division, Fondazione Toscana G. Monasterio, Pisa, Italy; 5 Cardiology, Cardiothoracic Department, Azienda Sanitaria Universitaria Friuli Centrale (ASUFC), Udine, Italy; 6 De Gasperis Cardio Center, Niguarda Hospital, Milan, Italy; 7 Faculty of Medicine, Imperial College London, National Heart & Lung Institute, London, UK; 8 Department of Cardiology, National Pulmonary Hypertension Service, Hammersmith Hospital, Imperial College NHS Trust, London, UK; 9 Division of Cardiology, Fondazione IRCCS Policlinico San Matteo, Pavia, Italy; 10 Department of Medicine, Rheumatology Clinic, Azienda Sanitaria Universitaria Friuli Centrale (ASUFC), Udine, Italy; 11 Department of Internal Medicine, University of Genova, Viale Benedetto XV, 6, 16132 Genoa, Italy; 12 Division of Cardiology, Department of Medicine, Karolinska Institutet, Stockholm, Sweden

**Keywords:** Pulmonary hypertension, Left heart disease, Comorbidity, Risk, Prognosis

## Abstract

**Aim:**

To obtain real-world evidence about the features and risk stratification of pulmonary arterial hypertension (PAH) with a left heart disease (LHD) phenotype (PAH-LHD).

**Methods and results:**

By reviewing the records of consecutive incident PAH patients at 7 tertiary centers from 2001 to 2021, we selected 286 subjects with all parameters needed to determine risk of death at baseline and at first follow-up with COMPERA and COMPERA 2.0 scores. Fifty seven (20%) had PAH-LHD according to the AMBITION definition. Compared with no-LHD ones, they were older, had higher BMI, more cardiovascular comorbidities, higher E/e’ ratio and left atrial area, but lower BNP concentrations and better right ventricular function and pulmonary hemodynamics. Survival was comparable between PAH-LHD and no-LHD patients, although the former were less commonly treated with dual PAH therapy. Both COMPERA and COMPERA 2.0 discriminated all-cause mortality risk of PAH-LHD at follow-up, but not at baseline. Risk profile significantly improved during follow-up only when assessed by COMPERA 2.0. At multivariable analysis with low-risk status as reference, intermediate-high and high-risk, but not LHD phenotype, were associated with higher hazard of all-cause mortality. Results were comparable in secondary analyses including patients in the last 10 years and atrial fibrillation and echocardiographic abnormalities as additional criteria for PAH-LHD.

**Conclusions:**

In real life, PAH-LHD patients are frequent, have less severe disease and are less likely treated with PAH drug combinations than no-LHD. The COMPERA 2.0 model may be more appropriate to evaluate their mortality risk during follow-up and how it is modulated by therapy.

**Supplementary Information:**

The online version contains supplementary material available at 10.1007/s00392-024-02448-9.

## Introduction

A growing number of patients diagnosed with pulmonary arterial hypertension (PAH) has features suggestive of concomitant left heart disease (LHD), albeit not clinically overt, nor sufficient to cause an elevation in pulmonary artery wedge pressure (PAWP) during rest right heart catheterization (RHC) beyond the 15-mmHg threshold, which would lead to diagnosis of group 2 pulmonary hypertension (PH) [1–3]. A LHD phenotype is more likely when established risk factors for LHD, including cardiovascular (CV) comorbidities, are present and when PAWP is at the upper limit of the normal range [4, 5].

In an analysis of the Comparative, Prospective Registry of Newly Initiated Therapies for Pulmonary Hypertension (COMPERA) registry, about 25% of patients with idiopathic PAH had ≥ 3 risk factors for LHD, namely arterial hypertension, diabetes mellitus (DM), body mass index (BMI) > 30 kg/m^2^, coronary artery disease (CAD), and atrial fibrillation (AF) [6]. The most accurate definition of PAH with a LHD phenotype (hereafter, PAH-LHD) has been proposed by the steering committee of the Study of First-Line Ambrisentan and Tadalafil Combination Therapy in Subjects With Pulmonary Arterial Hypertension (AMBITION) and includes both clinical and hemodynamic parameters [7]. A post-hoc study showed that 13.0% of the participants in the Prostacyclin (PGI2) Receptor Agonist In Pulmonary Arterial Hypertension (GRIPHON) phase 3 trial had PAH-LHD as per AMBITION criteria [8], but in the real-world scenario the prevalence of PAH-LHD has been reported to be > 30% [9].

According to current European Society of Cardiology (ESC) / European Respiratory Society (ERS) guidelines, therapeutic decisions in PAH should be based on the predicted risk of 1-year mortality [10]. Two risk assessment approaches have been developed by analyzing the COMPERA population, i.e. COMPERA [11] and COMPERA 2.0 [12]. The latter is based on readily available variables and provides a more granular risk stratification than the former, as well as than other risk estimates [13, 14]. The ESC/ERS guidelines recommend that COMPERA 2.0 should be used for risk re-evaluation during follow-up [10].

External validation of the COMPERA 2.0 has been attained in general PAH populations from registries, [15–18], but data about its performance in patients with PAH-LHD are lacking.

## Methods

### Study population

The study sample was drawn form a retrospective database of patients with PAH, followed between April 2001 and November 2021 at seven tertiary care centers (Trieste University Hospital, Trieste, Italy; Hammersmith Hospital, London, United Kingdom; IRCCS Ospedale Policlinico San Martino, Genova, Italy; University Hospital Spedali Civili of Brescia, Brescia, Italy; Fondazione G. Monasterio, Pisa, Italy; Niguarda Hospital, Milan, Italy; Udine University Hospital, Udine, Italy) [17].

PAH was diagnosed according to ESC/ERS guidelines and all patients underwent a complete diagnostic work-up to exclude PH of other groups. The hemodynamic cut-offs applied to define pre-capillary PH were mean pulmonary artery pressure (mPAP) ≥ 25 mmHg, PAWP ≤ 15 mmHg, and pulmonary vascular resistance (PVR) > 3 Wood units (WU). For the purpose of this analysis, we considered only patients with no missing information related to the variables required to perform risk stratification by the COMPERA and COMPERA 2.0 scores, both at diagnosis and within 12 months from diagnosis. Therefore, follow-up RHC was needed.

The study was approved by the institutional review board and was conducted in accordance with the ethical guidelines of the 1975 Declaration of Helsinki.

### Left heart disease phenotype

Patients were classified as having PAH-LHD according to the criteria established for the amendment of AMBITION [7], if they had: (i) more than 2 risk factors for left ventricular (LV) diastolic dysfunction between arterial hypertension, DM, BMI ≥ 30 kg/m^2^ or history of significant CAD (clinical criteria); or (ii) PVR between 3 and 3.75 WU, or PVR between 3.75 and 6.25 WU if PAWP was 13 to 15 mmHg (hemodynamic criteria) (Table 1). As per study design, none had PAWP ≥ 16 mmHg, i.e. group 2 PH.

**Table 1:** Criteria for definition of a left heart disease phenotype

Main analysis
Patients with at least one of the following:
(i) Clinical criteria:
≥ 3 of the following risk factors for LV diastolic dysfunction
- BMI ≥ 30 kg/m^2^
- history of systemic hypertension
- DM (any type)
- history of significant CAD
(ii) Hemodynamic criteria:
- PVR between 3 and 3.75 WU
- PVR between 3.75 to 6.25 WU in the presence of PAWP of 13 to 15 mmHg
Secondary analysis
Patients with at least one of the following:
(i) Clinical criteria:
(ia) ≥ 3 of the following risk factors for LV diastolic dysfunction:
- BMI ≥ 30 kg/m^2^
- history of systemic hypertension
- DM (any type)
- history of significant CAD
(ib): 2 of the above listed risk factors for LV diastolic dysfunction and ≥ 1 among:
- AF
- LVEF < 50%
- at least moderate mitral or aortic valve disease
- left atrial dilation
(ii) Hemodynamic criteria:
-PVR between 3 and 3.75 WU
-PVR between 3.75 to 6.25 WU in the presence of PAWP of 13 to 15 mmHg

*LV,* left ventricular; *BMI,* body mass index; *DM*, diabetes mellitus; *CAD,* coronary artery disease; *AF*, atrial fibrillation; *LVEF,* left ventricular ejection fraction; *PVR,* pulmonary vascular resistance; *PAWP,* pulmonary artery wedge pressure

As a secondary analysis, we expanded the clinical criteria suggestive for LHD, by including AF and echocardiographic signs of LHD (Table 1) [9].

### Risk assessment

The risk category at baseline and at the first follow-up was determined by calculating the COMPERA and COMPERA 2.0 scores (Supplementary Tables S1 and S2). For the former, we assigned 1 to 3 points (1 if low risk; 2 if intermediate risk; and 3 if high risk) to each of the following 5 variables, using the thresholds for low-, intermediate- and high-risk proposed by the 2015 ESC/ERS guidelines: World Health Organization Functional Class (WHO-FC), 6-min walking distance (6MWD), brain natriuretic peptide (BNP) or N-terminal pro-B-type natriuretic peptide (NT-proBNP), cardiac index (CI), and right atrial pressure (RAP). The mean score rounded to the next integer defined the patient global risk as low, intermediate, or high [11, 19].

For COMPERA 2.0, WHO-FC, 6MWD, and BNP or NT-proBNP were assigned a score between 1 and 4 according to the cut-offs set by the COMPERA investigators [12] and applied by the 2022 ESC/ERS guidelines [10]. The rounded mean score distinguished the patient risk status in low (mean score = 1), intermediate-low (mean score = 2), intermediate-high (mean score = 3), or high (mean score = 4).

### Statistical analysis

Normality was assessed with the Shapiro–Wilk test. Categorical and continuous variables are reported as count and percentage, mean ± standard deviation (SD) or median [interquartile range, IQR]. Patients’ characteristics were compared by chi-squared test, Fisher exact test, 2-sided student t test or Mann–Whitney test, as appropriate.

Kaplan–Meier curves for 7-years survival were estimated and compared across risk categories in the two groups of patients (no-LHD and LHD) using the log-rank test. Cox regression analysis was performed to assess the relationship between LHD status, risk strata and 7-year all-cause mortality.

The survival analyses were repeated after excluding patients with a PAH diagnosis before 2013, in order to limit the time frame of the analysis to a period during which the standards of PAH therapy had been similar.

P-values < 0.05 were considered significant. Statistical analyses were performed using R software (R version 3.6.1).

## Results

### Patient characteristics

Two-hundred eighty-six patients were included in the analysis, mostly females (69%) with a mean age of 58 ± 16 years and a diagnosis of idiopathic, hereditary, drug-induced or connective tissue disease-associated PAH (Table 2). Systemic sclerosis was the most common connective tissue disease (66 out of 91 subjects, 72.5%). Half of the patients were in WHO-FC III and median natriuretic peptide levels were high (948 ng/L for NT-proBNP and 253 pg/mL for BNP). After diagnosis, 40% of patients were treated with upfront dual oral combination therapy and the proportion rose to 55% during follow-up.

**Table 2:** Characteristics of the study population according to the main analysis criteria for a left heart disease phenotype

	Overall (N = 286)	no-LHD (N = 229)	PAH-LHD (N = 57)	P
Female	198 (69)	162 (71)	36 (63)	0.34
Age at diagnosis, years	58 ± 16	57 ± 16	65 ± 13	** < 0.001**
PAH classification				0.4
IPAH/HPAH/drug induced	139 (49)	117 (51)	22 (39)	
CTD	91 (32)	70 (31)	21 (37)	
PoPH	30 (10)	23 (10)	7 (12)	
Other	26 (9)	19 (8)	7 (12)	
BMI, Kg/m^2^	26.4 ± 6	25.7 ± 5.6	29.3 ± 6.5	** < 0.001**
SBP, mmHg	124 ± 18	122 ± 17	130 ± 18	**0.007**
DBP, mmHg	76 ± 12	76 ± 12	76 ± 10	0.98
WHO-FC				0.3
I	19 (7)	17 (8)	2 (4)	
II	84 (30)	63 (28)	21 (38)	
III	142 (51)	113 (50)	29 (52)	
IV	36 (13)	32 (14)	4 (7)	
HR, bpm	82 ± 16	83 ± 16	77 ± 13	**0.02**
Hypertension	119 (42)	83 (36)	36 (63)	** < 0.001**
CAD	38 (13)	20 (9)	18 (32)	** < 0.001**
Atrial fibrillation	41 (16)	27 (13)	14 (28)	0.01
VHD	33 (12)	24 (11)	9 (16)	0.35
Diabetes	56 (20)	31 (14)	25 (44)	** < 0.001**
CKD	59 (21)	44 (19)	15 (26)	0.32
Synus rhythm	262 (92)	215 (94)	47 (82)	**0.01**
DLCO, %	50 ± 20	50 ± 20	47 ± 20	0.48
6MWD, m	308 [192 – 408]	308 [192 – 408]	310 [240 – 405]	0.5
Haemoglobin, g/dL	13.9 ± 2.1	14.1 ± 2.2	13.3 ± 1.9	**0.01**
Creatinine, mg/dL	1.04 ± 0.54	1.01 ± 0.43	1.16 ± 0.83	0.22
NT-proBNP, ng/L	948 [261 – 2534]	1032 [281 – 2974]	447 [226 – 1415]	0.24
BNP, ng/L	253 [87 – 624]	320 [96 – 744]	126 [64 – 255]	**0.004**
Baseline echocardiography				
LVEF, %	59 ± 7	59 ± 7	60 ± 6	0.80
Mitral E peak, m/s	0.53 ± 0.24	0.51 ± 0.23	0.62 ± 0.28	**0.02**
Mitral E/A ratio	0.81 ± 0.44	0.81 ± 0.47	0.80 ± 0.23	0.14
Mitral E/e’ ratio	8.3 ± 4.5	7.8 ± 3.9	10.5 ± 5.8	** < 0.001**
LA area, cm^2^	18 ± 6	18 ± 6	21 ± 7	**0.001**
RV basal diameter, cm	4.8 [4.3 – 5.4]	4.8 [4.3 – 5.4]	4.8 [4.3 – 5.4]	0.92
TAPSE, mm	17 ± 8	17 ± 9	20 ± 5	** < 0.001**
FAC, %	28 ± 11	27 ± 10	34 ± 10	** < 0.001**
RVSP, mmHg	67 ± 22	69 ± 22	60 ± 19	**0.02**
eRAP, mmHg	8 ± 4	8 ± 4	6 ± 4	**0.03**
TAPSE/sPAP	0.26 ± 0.14	0.25 ± 0.13	0.33 ± 0.14	** < 0.001**
RVOT-AccT, ms	76 ± 20	75 ± 20	78 ± 21	0.41
RA area, cm^2^	24 ± 7	24 ± 7	23 ± 7	0.53
Pericardial effusion	79 (29)	66 (30)	13 (24)	0.50
Baseline right heart catheterization				
sPAP, mmHg	75 ± 21	78 ± 20	64 ± 20	** < 0.001**
dPAP, mmHg	31 ± 11	32 ± 11	24 ± 7	** < 0.001**
mPAP, mmHg	47 ± 13	49 ± 13	39 ± 11	** < 0.001**
PAWP, mmHg	9 ± 4	9 ± 3	12 ± 5	** < 0.001**
RAP, mmHg	8 ± 4	8 ± 5	8 ± 4	0.26
CO, L/min	4.21 ± 1.55	3.93 ± 1.31	5.33 ± 1.91	** < 0.001**
CI, L/min/m^2^	2.38 ± 0.81	2.27 ± 0.66	2.84 ± 1.13	** < 0.001**
PVR, WU	10.1 ± 5.57	11.1 ± 5.36	5.93 ± 4.39	** < 0.001**
Therapy after diagnosis				
ERA	174 (61)	144 (63)	30 (53)	0.21
PDE5i/GCs	192 (67)	161 (70)	31 (54)	**0.03**
Prostanoid	14 (5)	14 (6)	0	0.12
Dual oral	114 (40)	99 (43)	15 (26)	**0.03**
Beta-blockers	64 (22)	37 (16)	27 (47)	** < 0.001**
ACEi/ARB	71 (25)	48 (21)	23 (40)	**0.004**
Anticoagulation	80 (28)	64 (28)	16 (28)	1
Amiodarone	3 (1)	3 (1)	0	0.89
Digoxin	10 (4)	9 (4)	1 (2)	0.69
Therapy at first disease reassessment				
ERA	206 (72)	173 (76)	33 (58)	**0.008**
PDE5i/sGC	214 (75)	179 (78)	35 (61)	**0.009**
Prostanoid	40 (14)	37 (16)	3 (5)	**0.03**
Dual oral	156 (55)	136 (59)	20 (35)	** < 0.001**

Data are expressed as n (%), mean ± SD or median [IQR], as appropriate

*PAH,* pulmonary arterial hypertension; *IPAH*, idiopatic PAH; *HPAH*, hereditary PAH; *CTD*, connective tissue disease; *PoPH*, porto-pulmonary hypertension; *BMI,* body mass index; *SBP,* systolic blood pressure; *DBP,* diastolic blood pressure; *WHO-FC,* World Health Organization functional class; *HR*, heart rate; *CAD,* coronary artery disease; *VHD,* valvular heart disease; *CKD,* chronic kidney disease; *DLCO,* diffusion capacity of carbon monoxide; *6MWD*, six-minute walking distance; *LVEF*, left ventricular ejection fraction; *LA*, left atrium; *RV*, right ventricle; *TAPSE,* tricuspid annular plane systolic excursion; *FAC*, fractional area change; *RVSP,* right ventricular systolic pressure; *eRAP*, estimated right atrial pressure; *RVOT-AccT*, right ventricle outflow tract acceleration time; *RA,* right atrium; *mPAP, dPAP and sPAP* for mean, diastolic and systolic pulmonary artery pressure; *PAWP,* pulmonary artery wedge pressure; *RAP,* right atrial pressure; CO, cardiac output; CI, cardiac index; *PVR,* pulmonary vascular resistance; *ERA,* endothelin receptor antagonist; *PDE5i,* phosphodiesterase type 5 inhibitor; *GCs,* guanylate cyclase stimulator; *ACEi,* angiotensin converting enzyme inhibitors*; ARB,* angiotensin receptor blocker

Fifty-seven (20%) patients had PAH-LHD according to the AMBITION criteria (Table 2). Of them, 16 had clinical features and 34 had hemodynamic features of PAH-LHD; only 7 fulfilled both clinical and hemodynamic criteria. When AF was added to the risk factors for LHD, 37 (13%) patients had clinical characteristics of PAH-LHD.

Subjects with PAH-LHD were older, had higher BMI and more often CV comorbidities than no PAH-LHD ones. They had better parameters of right ventricular (RV) function and pulmonary hemodynamics; however, they had worse measures of LV diastolic function, i.e. E/e’ ratio, left atrial area, and PAWP. BNP concentrations were lower in the LHD than no-LHD group (Table 2). Vasoreactivity testing was positive in 28 patients: 21 (9.3%) without and 7 (12.3%) with PAH-LHD (P = 0.50). A fluid challenge had not been performed in any patient.

Subjects with PAH-LHD were less commonly treated with phosphodiesterase type 5 inhibitors/guanylate cyclase stimulators and dual oral therapy, while they were more often prescribed with beta-blockers and angiotensin-converting enzyme inhibitors or angiotensin receptor blockers, as expected considering their comorbidities.

**Fig. 1:**
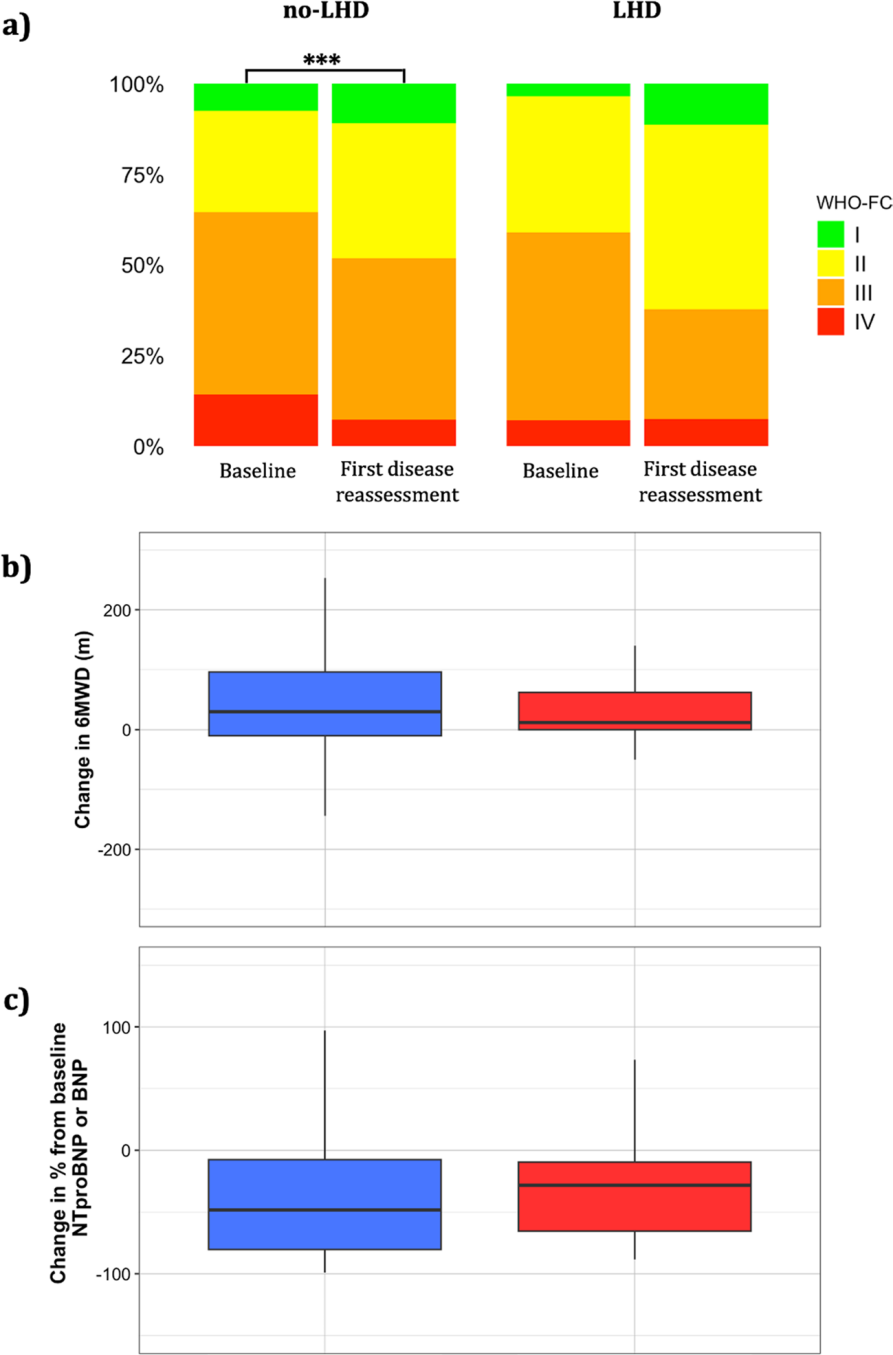
Changes in World Health Organization functional class (a), six-minute walking distance (b) and natriuretic peptide concentrations (c) among patients with (red in panels b and c) and without (blue in b and c) a left heart disease (LHD) phenotype. *** indicates P < 0.001

The first disease reassessment including RHC was done 7 ± 5 months after diagnosis. The distribution of WHO-FC significantly changed only in patients without a LHD phenotype (Fig. 1), but the two groups had similar rates of ≥ 1 class improvement (31% in no-LHD and 38% in LHD, P = 0.30). Changes in 6MWD (+ 30 [-10; + 96] vs + 12 [0; + 62] meters, P = 0.71) and in natriuretic peptide concentrations (-46 [-79; -2] % vs –26 [-64; + 9] %, P = 0.08) were numerically, but not significantly, greater in the no-LHD group (Fig. 1).

### Risk assessment

At baseline, risk distribution was significantly different between patients with vs without a LHD phenotype according to the COMPERA model, with more subjects with PAH-LHD being at low risk and less at high risk (P = 0.03). By contrast, no difference was found with the COMPERA 2.0 model (P = 0.4).

**Fig. 2:**
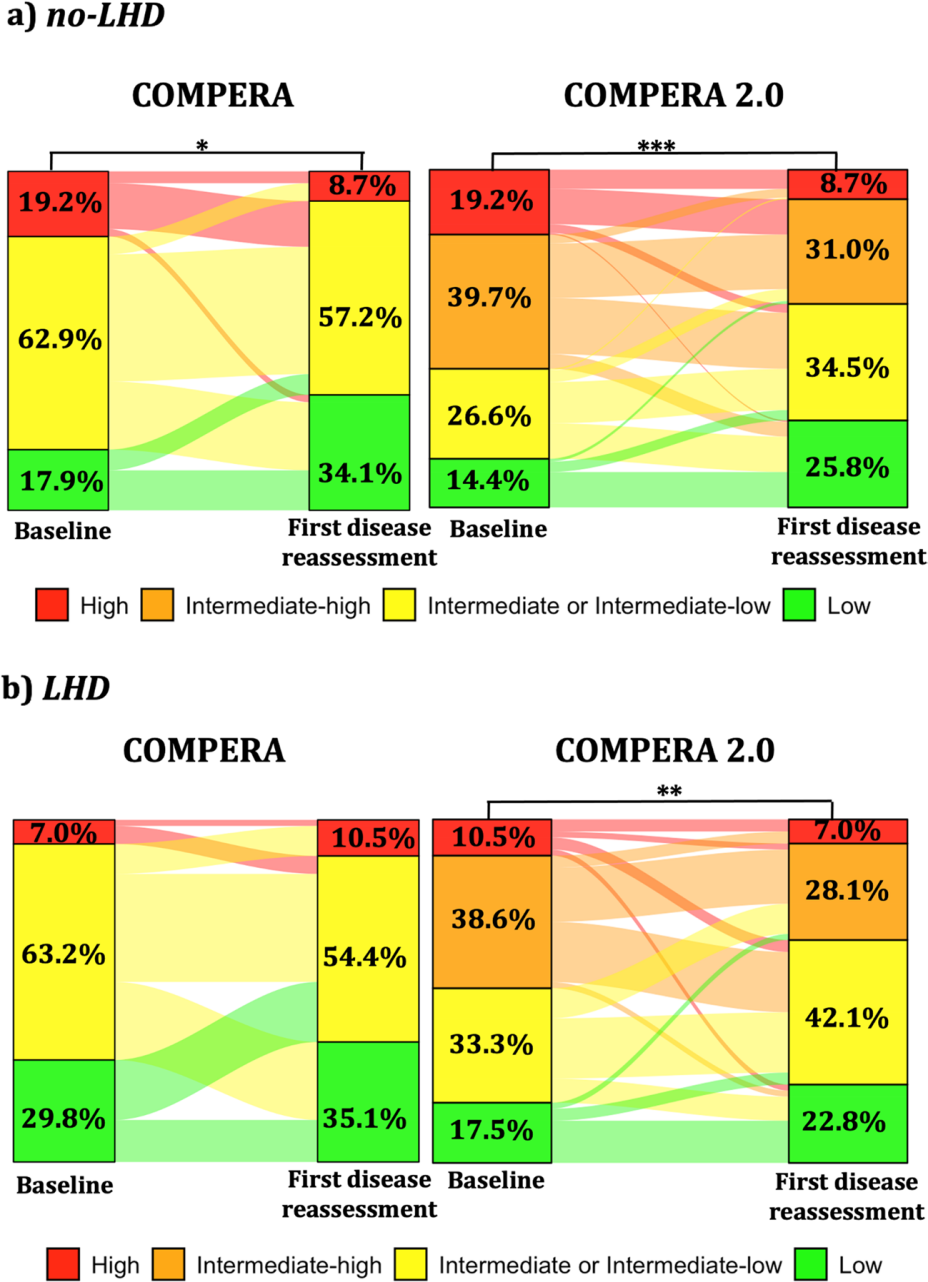
Changes in risk categories from baseline to first disease reassessment according to the COMPERA and COMPERA 2.0 models in patients without (a) and with (b) a left heart disease (LHD) phenotype according to the main analysis criteria. *, ** and *** indicate P < 0.05, P < 0.01 and P < 0.001, respectively

Irrespective of the presence of a LHD phenotype, the majority of patients were classified at intermediate risk with the COMPERA score (Fig. 2). Using the COMPERA score, a significant reduction in the proportion of subjects at high or intermediate risk was observed only for the no-LHD group. Conversely, when the COMPERA 2.0 score was calculated, risk improvement was significant in both groups, with 60.3% and 64.9%, respectively, reaching the low or intermediate-low risk status (Fig. 2).

### Survival

The median follow-up duration was 2.9 [1.8–4.7] years for patients without a LHD profile and 2.8 [1.0–4.9] years for those with PAH-LHD (P = 0.39). The Kaplan–Meier estimated survival rates at 1, 3 and 5 years were 96%, 82% and 65%, and 96%, 88% and 76%, respectively (log-rank P = 0.30). Within each risk stratum, as calculated by either the COMPERA or the COMPERA 2.0 approach, survival was comparable between patients with or without a LHD phenotype (Supplementary Fig. S1-S4).

**Fig. 3:**
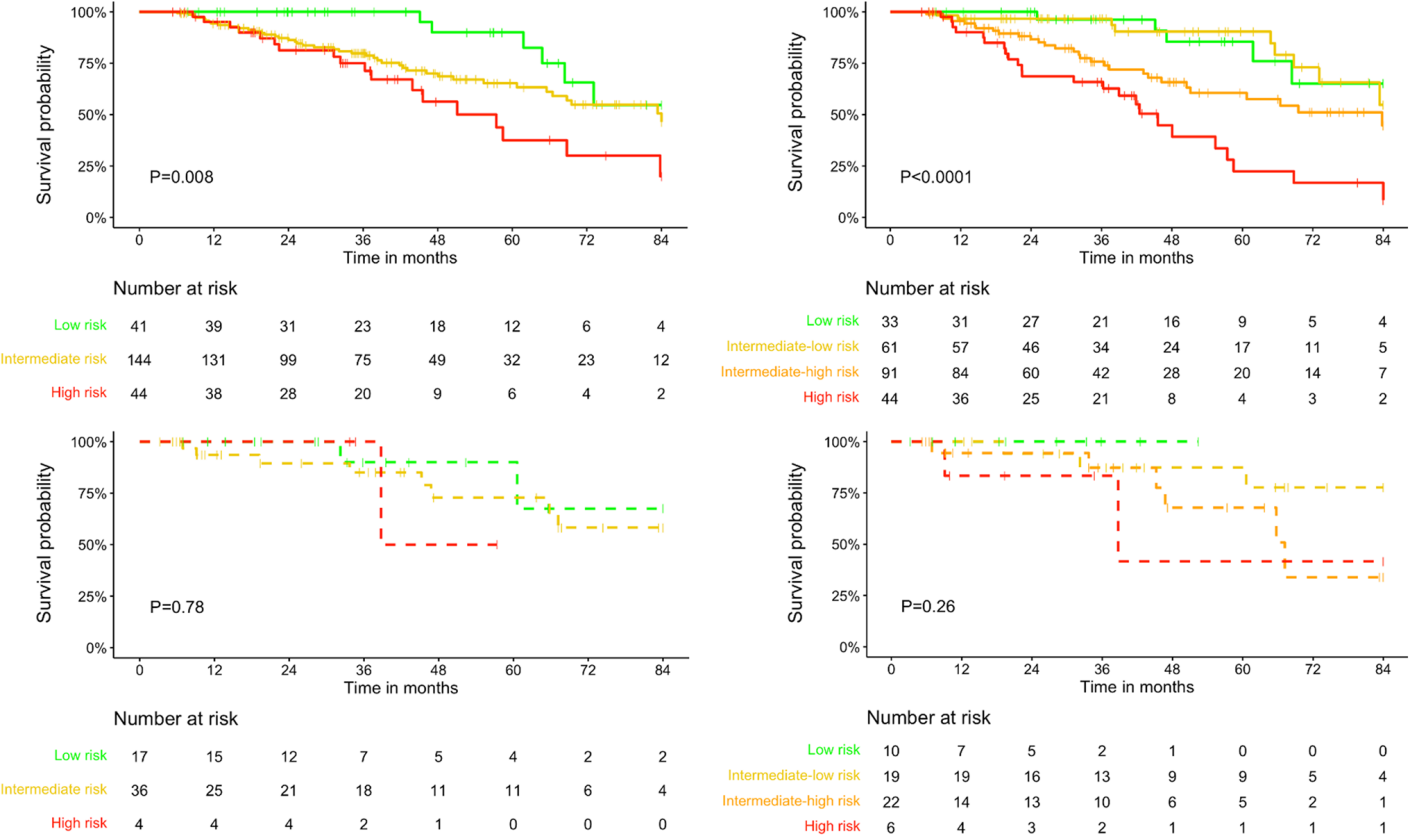
Survival curves according to risk strata at baseline, as assessed by the COMPERA (left) and COMPERA 2.0 (right) models, in patients with (dashed lines) and without (solid lines) a left heart disease (LHD) phenotype

**Fig. 4:**
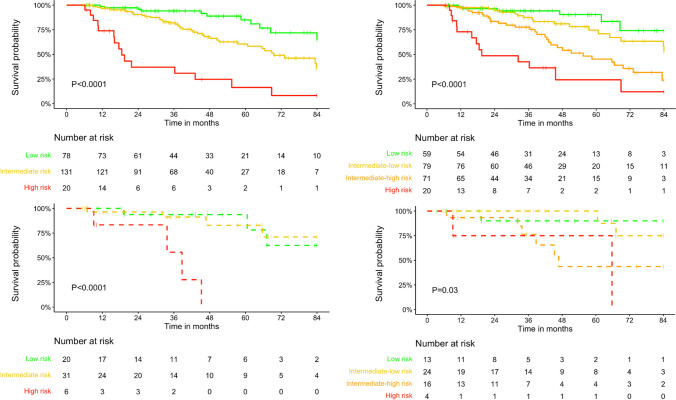
Survival curves according to risk strata at first disease reassessment, as assessed by the COMPERA (left) and COMPERA 2.0 (right) models, in patients with (dashed lines) and without (solid lines) a left heart disease (LHD) phenotype

Survival curves according to risk status at diagnosis and first disease reassessment based on the COMPERA and COMPERA 2.0 scores are shown in Fig. 3 and Fig. 4. While all-cause mortality was significantly different across baseline COMPERA and COMPERA 2.0 risk strata in no-LHD, either method for risk stratification failed to discriminate the prognosis in PAH-LHD risk subgroups. However, both COMPERA and COMPERA 2.0 risk categories differentiated mortality at first disease revaluation regardless of the presence of a LHD phenotype.

At Cox regression analysis, higher risk status, but not LHD, was associated with increased all-cause mortality (Table 3). Remarkably, intermediate-low risk, as determined by COMPERA 2.0, both at baseline and first reassessment was not associated with reduced survival as compared with low-risk status.

**Table 3:** Cox regression analysis evaluating the association of risk status and a left heart disease phenotype (main analysis criteria) with 7-year all-cause mortality

	HR	95% IC	P
LHD vs no-LHD	0.69	0.36 – 1.30	0.25
Baseline COMPERA risk (vs low risk)			
Intermediate risk	2.22	1.05 – 4.67	**0.04**
High risk	3.80	1.66 – 8.70	**0.002**
COMPERA risk at first reassessment (vs low risk)			
Intermediate risk	2.27	1.25 – 4.14	**0.007**
High risk	10.99	5.48 – 22.03	** < 0.001**
Baseline COMPERA 2.0 risk (vs low risk)			
Intermediate-low	1.16	0.41 – 3.29	0.78
Intermediate-high	2.94	1.15 – 7.51	**0.02**
High	6.30	2.41 – 16.43	** < 0.001**
COMPERA 2.0 risk at first reassessment (vs low risk)			
Intermediate-low	1.74	0.73 – 4.11	0.21
Intermediate-high	4.51	2.01 – 10.14	** < 0.001**
High	11.17	4.54 – 27.44	** < 0.001**

### Secondary analyses

By definition, more patients (80, 28%) had PAH-LHD in the secondary analysis with expanded clinical criteria for a LHD phenotype (Supplementary Table S3). Again, most had either clinical (n = 39) or hemodynamic (n = 29) criteria for PAH-LHD, with only 12 subjects presenting both.

The comparison between the groups with and without a LHD phenotype was consistent with the main analysis. Like in this latter, a significant improvement in WHO-FC was only found in no-LHD patients, while changes in natriuretic peptides or 6MWD were not significantly different between the LHD and no-LHD groups (Supplementary Fig. S5).

A significant change in the distribution of risk classes from baseline to follow-up was observed only with COMPERA 2.0 (Supplementary Fig. S6). Survival analyses were consistent with those of the main analysis (Supplementary Fig. S7 and S8).

The characteristics of patients with vs without a LHD phenotype were also alike in the main analysis when only patients from 2013 to 2021 were considered (Supplementary Table S4), as was the survival analysis by COMPERA strata (Supplementary Fig. S9 and S10).

## Discussion

In this multicentric cohort followed at tertiary centers in Italy and UK for the last 20 years, 1 in 5 patients with PAH had features suggestive of concealed LHD. Although most often treated with single therapy, they had a survival similar to those without features of LHD. In these subjects, both the COMPERA and COMPERA 2.0 tools performed better in predicting prognosis at follow-up than at baseline. However, only COMPERA 2.0 reflected the effect of therapy by reclassifying a significant proportion of patients into the low and intermediate-low risk strata.

The age of the PAH population has progressively increased worldwide. Mean age of PAH patients in the Swiss PH registry rose from 53 ± 16 years between 2000 and 2004 to 60 ± 15 years between 2009 and 2012 [20]. Both the COMPERA registry and the Registry to Evaluate Early and Long-term PAH Disease Management (REVEAL), 2 large-scale ongoing observational studies in Europe and USA, now include sizable numbers of old individuals with PAH [21, 22].

This demographic shift explains why risk factors for LHD have become common among PAH patients. By convention, LHD is considered likely when at least 3 diseases are present among the following: arterial hypertension, DM, obesity, and CAD. AF is also often taken into account, as it is mostly associated with LHD. The frequency of patients with such a clinical profile has been shown to range from approximately 16% to 25% [6, 9, 23].

It should be highlighted that having conditions that predispose to LV diastolic dysfunction does not necessarily implicate elevated left heart filling pressures at rest. Pulmonary hemodynamics may be indicative of combined post- and pre-capillary PH, rather than of pre-capillary PH, even if the aforementioned risk factors for LHD do not cluster in one patient. Likewise, a classical PAH hemodynamic profile, with high PVR and low PAWP, may be observed in spite of concomitant comorbidities heralding LHD. This consideration is buttressed by the observation that a minority of the patients in our registry had coexistence of clinical and hemodynamic criteria for occult LHD (7 out of 57, 12.3% in the main analysis; and 12 out of 80, 15% in the secondary analysis). A similar finding was made in the post-hoc analysis of the GRIPHON trial, in which 55.6% met the clinical definition of PAH-LHD, 39.4% the hemodynamic one, and just 5% both [8]. In an Italian study of elderly PAH patients, there was even no overlap between clinical or hemodynamic criteria for PAH-LHD [9].

Being the classification of PAH-LHD quite discordant by means of clinical or hemodynamic parameters, the combination of either type of information, as adopted by the AMBITION steering committee, appears to be the most effective way not to miss subjects with PAH-LHD. To the best of our knowledge, this is the first report of the real-life frequency of PAH-LHD as per AMBITION criteria. The prevalence we found (20%) is higher than the one described in the GRIPHON trial (13%); however, enrollment in the latter was not allowed if PVR was < 5 WU or age was > 75 years, thus favoring the selection of individuals without PAH-LHD.

Compared with those without LHD, the patients with PAH-LHD in this study had signs of LV diastolic dysfunction, indicating that adopted definition reliably identifies PAH-LHD. They also had milder PH and RV impairment with lower concentrations of BNP and, although not to a significant extent, NT-proBNP. Nonetheless, functional status, as determined by WHO-FC class and 6MWD, was alike in the no-LHD and PAH-LHD groups. This finding is in contrast with previous investigations, which related higher symptom burden and worse functional capacity to CV comorbidities [22–26], possibly because the patients without a LHD phenotype in our cohort had more advanced PAH. Modulation of natriuretic peptide levels by neurohormonal inhibitors in subjects with PAH-LHD is also possible and may have influenced the differences as compared with those without a LHD phenotype.

The principles of PAH treatment have evolved in the long time period covered by this analysis, justifying the relatively low use of 2 oral drugs we found at both diagnosis and follow-up. With this shortcoming acknowledged, it is notable that dual therapy in patients with PAH-LHD was almost half as frequent as in patients without a LHD phenotype. This prescription pattern has already been noted by other authors and is supported by current guidelines, which recommend that treatment of PAH with comorbidities should be cautious and start with 1 drug [10, 24–27].

However, there may be patients with PAH-LHD who benefit from combination therapy. In the GRIPHON trial, the reduction in the risk of a morbidity/mortality event attained with selexipag was consistent in participants fulfilling or not the AMBITION definition of PAH-LHD, and 80% were already on an endothelin receptor antagonist and/or a phosphodiesterase type 5 inhibitor at the time of randomization, with 30% on dual treatment [8]. In a recent, single-center study from the Netherlands, the rate of dual therapy and the subsequent hemodynamic and functional response were not different between PAH patients with vs without a high probability of heart failure with preserved ejection fraction according to the H2FPEF score [28].

In our cohort, the improvement in WHO-FC, 6MWD, and NT-proBNP/BNP levels was somehow smaller in patients with than without a LHD phenotype, but survival was comparable, suggesting that a less aggressive therapy in PAH-LHD might be appropriate.

Stratification of the risk of 1-year mortality is now mandatory in PAH management, since treatment intensity and subsequent escalation are based on predicted survival in the current therapeutic algorithm [10, 29]. While a comprehensive assessment incorporating clinical, functional, imaging, and hemodynamic variables is preferred at baseline, a simplified approach including a limited number of measurements is accepted for long-term follow-up [10, 30–32]. Most proposed tools for streamlined evaluation of risk in PAH distinguish 3 profiles with increasingly worse prognosis: low, intermediate, and high [11, 14, 33]. The majority of patients are classified at intermediate risk despite having various severity of disease; thereby, the treatment generically advised for the broad intermediate-risk category may not always be suitable. This flaw is overcome by the COMPERA 2.0 model, which further divides the intermediate-risk class in intermediate-low and intermediate-high. Furthermore, the COMPERA 2.0 4-strata model is more sensitive to modifications of risk from baseline to follow-up than other models [12].

CV comorbidities have already been shown to negatively influence the performance of PAH risk scores at baseline [24, 25, 27]. In our study COMPERA and COMPERA 2.0 failed to stratify the risk of all-cause mortality in patients with PAH-LHD at baseline, but showed good discrimination capacity at follow-up. Importantly, treatment of PAH-LHD resulted in a significantly greater proportion of subjects with intermediate-low or low risk at first disease reassessment. This is clinically meaningful, since the intermediate-low and low risk classes were associated with better survival in multivariable analysis. It is also noteworthy that a LHD phenotype did not portend an increased risk of death after multiple adjustment.

Overall, these data validate the use of COMPERA 2.0 for follow-up risk re-estimation in the difficult setting of PAH-LHD. Interestingly, in the aforementioned Dutch investigation, the percentage of PAH patients in the COMPERA 2.0 intermediate-low and low risk categories increased from baseline to follow-up regardless of the H2FPEF score [28].

The present work has limitations. Risk stratification in PAH was implemented in the last years of the study period, as were some medications. Nonetheless, risk assessment at follow-up by COMPERA 2.0 was effective, confirming the usability of this tool even in patients not managed according to the standards of care for PAH. Furthermore, the results of a sensitivity analysis focusing on patients enrolled from 2013 onwards were in the same direction as those of the main analysis. Second, our cohort was selected based on the previous hemodynamic definition of PAH, instead of that given by the most recent guidelines. We decided to do so to facilitate the interpretation of the results of this analysis in the light of other ones, as the cut-offs of mPAP ≥ 25 mmHg, PAWP ≤ 15 mmHg, and PVR > 3 WU have been used in all other studies conducted so far to validate risk scores in PAH. Third, an exaggerated response to rapid infusion of saline may be another tool to unmask LHD in patients with PAWP ≤ 15 mmHg, but it was not evaluated in the patient sample we analysed. Fourth, the inclusion of patients with RHC at follow-up may have led to an underestimation of the frequency of PAH-LHD.

## Conclusions

The prevalence of PAH-LHD is high in tertiary PH centers. This PAH phenotype tends to be captured by either clinical or hemodynamic criteria, is associated with less severe vascular remodeling and RV impairment, and is most commonly treated with single PAH therapy. Nonetheless, long-term survival is comparable to that of PAH without concealed LHD. The 4-strata COMPERA 2.0 model allows adequately stratifying patients with PAH-LHD during follow-up.

## Supplementary Information

Below is the link to the electronic supplementary material.

Supplementary file1 (DOC 2.91 MB)

## Data Availability

Data will be made available by the corresponding authors upon reasonable request.

## References

[1] Marra AM, Benjamin N, Cittadini A, Bossone E, Grünig E (2022) When pulmonary hypertension complicates heart failure. Cardiol Clin 40(2):191–198. 10.1016/j.ccl.2021.12.007 10.1016/j.ccl.2021.12.007 35465893

[2] Hoeper MM, Gibbs JSR (2014) The changing landscape of pulmonary arterial hypertension and implications for patient care. Eur Respir Rev 23(134):450–457. 10.1183/09059180.00007814 25445943 10.1183/09059180.00007814 PMC9487398

[3] Lang IM, Palazzini M (2019) The burden of comorbidities in pulmonary arterial hypertension. Eur Heart J Suppl 21(Suppl K):K21–K28. 10.1093/eurheartj/suz205 31857797 10.1093/eurheartj/suz205 PMC6915052

[4] Hoeper MM, Dwivedi K, Pausch C et al (2022) Phenotyping of idiopathic pulmonary arterial hypertension: a registry analysis. Lancet Respir Med 10(10):937–948. 10.1016/S2213-2600(22)00097-2 35777416 10.1016/S2213-2600(22)00097-2 PMC9514996

[5] Vachiéry JL, Tedford RJ, Rosenkranz S et al (2019) Pulmonary hypertension due to left heart disease. Eur Respir J 53(1):1801897. 10.1183/13993003.01897-2018 30545974 10.1183/13993003.01897-2018 PMC6351334

[6] Opitz CF, Hoeper MM, Gibbs JSR, Kaemmere H, Pepke-Zaba J, Coghlan JG et al (2016) Pre-Capillary, Combined, and Post-Capillary Pulmonary Hypertension: A Pathophysiological Continuum. J Am Coll Cardiol 68(4):368–378. 10.1016/j.jacc.2016.05.047 27443433 10.1016/j.jacc.2016.05.047

[7] Galiè N, Barberà JA, Frost AE et al (2015) Initial Use of Ambrisentan plus Tadalafil in Pulmonary Arterial Hypertension. N Engl J Med 373(9):834–844. 10.1056/NEJMoa1413687 26308684 10.1056/NEJMoa1413687

[8] Rosenkranz S, Channick R, Chin KM, Jenner B, Gaine S, Galiè N et al (2022) The impact of comorbidities on selexipag treatment effect in patients with pulmonary arterial hypertension: insights from the GRIPHON study. Eur J Heart Fail 24(1):205–214. 10.1002/ejhf.2369 34806261 10.1002/ejhf.2369 PMC9298818

[9] Toma M, Miceli R, Bonsante E, Colombo D, Confalonieri M, Garascia A et al (2022) Left Heart Disease Phenotype in Elderly Patients with Pulmonary Arterial Hypertension: Insights from the Italian PATRIARCA Registry. J Clin Med 11(23):7136. 10.3390/jcm11237136 36498710 10.3390/jcm11237136 PMC9735657

[10] Humbert M, Kovacs G, Hoeper MM et al (2022) 2022 ESC/ERS Guidelines for the diagnosis and treatment of pulmonary hypertension. Eur Heart J 43(38):3618–3731. 10.1093/eurheartj/ehac237 36017548 10.1093/eurheartj/ehac237

[11] Hoeper MM, Kramer T, Pan Z et al (2017) Mortality in pulmonary arterial hypertension: prediction by the 2015 European pulmonary hypertension guidelines risk stratification model. Eur Respir J 50:1700740. 10.1183/13993003.00740-2017 28775047 10.1183/13993003.00740-2017

[12] Hoeper MM, Pausch C, Olsson KM et al. (2022) COMPERA 2.0: A refined four-stratum risk assessment model for pulmonary arterial hypertension. Eur Respir J. Jul 7;60(1):2102311. 10.1183/13993003.02311-2021 10.1183/13993003.02311-2021 PMC9260123 34737226

[13] Kylhammar D, Kjellström B, Hjalmarsson C et al (2018) A comprehensive risk stratification at early follow-up determines prognosis in pulmonary arterial hypertension. Eur Heart J 39(47):4175–4181. 10.1093/eurheartj/ehx257 28575277 10.1093/eurheartj/ehx257

[14] Dardi F, Manes A, Guarino D, Zuffa E, De Lorenzis A, Magnani I et al (2021) A pragmatic approach to risk assessment in pulmonary arterial hypertension using the 2015 European Society of Cardiology/European Respiratory Society guidelines. Open Heart 8(2):e001725. 10.1136/openhrt-2021-001725 34667092 10.1136/openhrt-2021-001725 PMC8527122

[15] Vicaire H, Le Pavec J, Mercier O, Montani D, Boucly A, Roche A et al (2022) Risk stratification in patients with pulmonary arterial hypertension at the time of listing for lung transplantation. J Heart Lung Transplant 41(9):1285–1293. 10.1016/j.healun.2022.06.001 35778258 10.1016/j.healun.2022.06.001

[16] Cruz-Utrilla A, Gallego-Zazo N, Pérez-Olivares C, Hernández-González I, Bedate P, MartínezMeñaca A et al (2023) Usefulness of genetics for clinical reclassification and refinement of prognostic stratification in pulmonary arterial hypertension. Rev Esp Cardiol (Engl Ed) 76(6):460–467. 10.1016/j.rec.2022.11.002 36403940 10.1016/j.rec.2022.11.002

[17] Stolfo D, Barbisan D, Ameri P, Lombardi CM, Monti S, Driussi M et al (2023) Performance of risk stratification scores and role of comorbidities in older vs younger patients with pulmonary arterial hypertension. J Heart Lung Transplant 42(8):1082–1092. 10.1016/j.healun.2023.02.1707 37005100 10.1016/j.healun.2023.02.1707

[18] Fadah K, Rodriguez JBC, Alkhateeb H, Mukherjee D, Garcia H, Schuller D et al (2023) Prognosis in Hispanic patient population with pulmonary arterial hypertension: An application of common risk stratification models. Pulm Circ 13(2):e12209. 10.1002/pul2.12209 37020706 10.1002/pul2.12209 PMC10069240

[19] Galiè N, Humbert M, Vachiery JL et al (2016) 2015 ESC/ERS Guidelines for the diagnosis and treatment of pulmonary hypertension: The Joint Task Force for the Diagnosis and Treatment of Pulmonary Hypertension of the European Society of Cardiology (ESC) and the European Respiratory Society (ERS): Endorsed by: Association for European Paediatric and Congenital Cardiology (AEPC), International Society for Heart and Lung Transplantation (ISHLT). Eur Heart J 37(1):67–119. 10.1093/eurheartj/ehv317 26320113 10.1093/eurheartj/ehv317

[20] Mueller-Mottet S, Stricker H, Domenighetti G et al (2015) Long-term data from the Swiss pulmonary hypertension registry. Respiration 89(2):127–140. 10.1159/000370125 25661477 10.1159/000370125

[21] Badesch DB, Raskob GE, Elliott CG et al (2010) Pulmonary arterial hypertension: baseline characteristics from the REVEAL Registry. Chest 137(2):376–387. 10.1378/chest.09-1140 19837821 10.1378/chest.09-1140

[22] Hoeper MM, Huscher D, Ghofrani HA et al (2013) Elderly patients diagnosed with idiopathic pulmonary arterial hypertension: results from the COMPERA registry. Int J Cardiol 168(2):871–880. 10.1016/j.ijcard.2012.10.026 23164592 10.1016/j.ijcard.2012.10.026

[23] Arvanitaki A, Vrana E, Boutsikou M et al (2022) The impact of cardiovascular comorbidities associated with risk for left heart disease on idiopathic pulmonary arterial hypertension: Data from the Hellenic Pulmonary Hypertension Registry (HOPE). Pulm Circ 12(2):e12086. 10.1002/pul2.12086 35685948 10.1002/pul2.12086 PMC9171835

[24] Bouzina H, Rådegran G, Butler O et al (2021) Longitudinal changes in risk status in pulmonary arterial hypertension. ESC Heart Fail 8(1):680–690. 10.1002/ehf2.13162 33305545 10.1002/ehf2.13162 PMC7835578

[25] Badagliacca R, D’Alto M, Ghio S et al (2022) Relevance of comorbidities on initial combination therapy in pulmonary arterial hypertension. ERJ Open Res 8(4):00298–02022. 10.1183/23120541.00298-2022 36382240 10.1183/23120541.00298-2022 PMC9638831

[26] Hoeper MM, Pausch C, Grünig E et al (2020) Idiopathic pulmonary arterial hypertension phenotypes determined by cluster analysis from the COMPERA registry. J Heart Lung Transplant 39(12):1435–1444. 10.1016/j.healun.2020.09.011 33082079 10.1016/j.healun.2020.09.011

[27] Charalampopoulos A, Howard LS, Tzoulaki I et al (2014) Response to pulmonary arterial hypertension drug therapies in patients with pulmonary arterial hypertension and cardiovascular risk factors. Pulm Circ 4(4):669–678. 10.1086/678512 25610602 10.1086/678512 PMC4278626

[28] Kianzad A, van Wezenbeek J, Celant LR et al (2022) Idiopathic pulmonary arterial hypertension patients with a high H2FPEF-score: Insights from the Amsterdam UMC PAH-cohort. J Heart Lung Transplant 41(8):1075–1085. 10.1016/j.healun.2022.05.007 35697604 10.1016/j.healun.2022.05.007

[29] Benza RL, Gomberg-Maitland M, Farber HW et al (2022) Contemporary risk scores predict clinical worsening in pulmonary arterial hypertension - An analysis of FREEDOM-EV. J Heart Lung Transplant 41(11):1572–1580. 10.1016/j.healun.2022.08.006 36117055 10.1016/j.healun.2022.08.006

[30] Galiè N, Channick RN, Frantz RP et al (2019) Risk stratification and medical therapy of pulmonary arterial hypertension. Eur Respir J 53(1):1801889. 10.1183/13993003.01889-2018 30545971 10.1183/13993003.01889-2018 PMC6351343

[31] Weatherald J, Boucly A, Sitbon O (2018) Risk stratification in pulmonary arterial hypertension. Curr Opin Pulm Med 24(5):407–415. 10.1097/MCP.0000000000000510 30004992 10.1097/MCP.0000000000000510

[32] Weatherald J, Boucly A, Sahay S, Humbert M, Sitbon O (2018) The Low-Risk Profile in Pulmonary Arterial Hypertension. Time for a Paradigm Shift to Goal-oriented Clinical Trial Endpoints. Am J Respir Crit Care Med 197(7):860–868. 10.1164/rccm.201709-1840PP 29256625 10.1164/rccm.201709-1840PP

[33] Boucly A, Weatherald J, Savale L et al (2017) Risk assessment, prognosis and guideline implementation in pulmonary arterial hypertension. Eur Respir J 50(2):1700889. 10.1183/13993003.00889-2017 28775050 10.1183/13993003.00889-2017

