# Supplementary material for: Frequency, characteristics and risk assessment of pulmonary arterial hypertension with a left heart disease phenotype
Source: Clin Res Cardiol. 2024 Apr 15;114(2):215–26. doi: 10.1007/s00392-024-02448-9 (PMC11839797; doi:10.1007/s00392-024-02448-9)

**Frequency, characteristics and risk assessment of pulmonary arterial hypertension with a left heart disease phenotype**

Matteo Toma, Giulio Savonitto, Carlo Maria Lombardi, Edoardo Airò, Mauro Driussi, Piero Gentile, Luke Howard, Martina Moschella, Emma Di Poi, Matteo Pagnesi, Simonetta Monti, Valentino Collini, Luciana D’Angelo, Veronica Vecchiato, Alberto Giannoni, Marianna Adamo, Davide Barbisan, Carolina Bauleo, Andrea Garascia, Marco Metra, Gianfranco Sinagra, Francesco Lo Giudice, Davide Stolfo, Pietro Ameri

**SUPPLEMENTARY APPENDIX**

**Table S1.** Risk stratification according to the COMPERA score.

**Table S2.** Risk stratification according to the COMPERA 2.0 score.

**Table S3**. Characteristics of the study population according to the secondary analysis criteria for a left heart disease (LHD) phenotype.

**Table S3**. Characteristics of the study population, as categorized by the presence of a LHD phenotype, when limited to patients with a diagnosis of PAH from 2013 to 2021.

**Figure S1:** Survival curves in patients without and with a LHD phenotype for each COMPERA risk stratum at baseline.

**Figure S2:** Survival curves in patients without and with a LHD phenotype for each COMPERA risk stratum at first disease reassessment.

**Figure S3.** Survival curves in patients without and with a LHD phenotype for each COMPERA 2.0 risk stratum at baseline.

**Figure S4.** Survival curves in patients without and with a LHD phenotype for each COMPERA 2.0 risk stratum at first disease reassessment.

**Figure S5**. Changes in World Health Organization functional class (a), six-minute walking distance (b) and natriuretic peptide concentrations (c) in patients with (red) and without (blue) a LHD phenotype according to the secondary analysis criteria.

**Figure S6**. Changes in risk from baseline to first disease reassessment according to COMPERA and COMPERA 2.0 models in patients without (a) and with (b) a LHD phenotype according to the secondary analysis criteria.

**Figure S7**. Survival curves according to risk strata at baseline, as assessed by baseline the COMPERA and COMPERA 2.0 models, in patients with (dashed lines) and without (solid lines) a LHD phenotype according to the secondary analysis criteria.

**Figure S8**. Survival curves according to risk strata at first disease reassessment, as assessed by the COMPERA and COMPERA 2.0 models, in patients with (dashed lines) and without (solid lines) a LHD phenotype according to the secondary analysis criteria.

**Figure S9**. Survival curves according to risk strata at baseline, as assessed by baseline the COMPERA and COMPERA 2.0 models, in patients diagnosed between 2013 and 2021 with (dashed lines) and without (solid lines) a LHD.

**Figure S10**. Survival curves according to risk strata at first disease reassessment, as assessed by the COMPERA and COMPERA 2.0 models, in patients diagnosed between 2013 and 2021 with (dashed lines) and without (solid lines) a LHD phenotype.

**Table S1**. Risk stratification according to the COMPERA score.

|  | Points assigned | | |
| --- | --- | --- | --- |
|  | 1 | 2 | 3 |
| WHO-FC | I or II | III | IV |
| 6MWD, m | >440 | 165 – 440 | <165 |
| BNP, ng/L | <50 | 50 – 300 | >300 |
| NTproBNP ng/L | <300 | 300 – 1400 | >1400 |
| RAP, mmHg | <8 | 8 – 14 | >14 |
| CI, L/min/m^2^ | ≥2.5 | 2.0 – 2.4 | <2.0 |
| Total score | = mean score (rounded to the next integer) | | |
|  | 1 | 2 | 3 |
| Risk category | Low | Intermediate | High |

*WHO-FC*, World Health Organization functional class; *6MWD*, six-minute walking diastance; *BNP and NTproBNP*, brain natriuretic peptide; *RAP*, right atrial pressure; *CI*, cardiac index.

**Table S2**. Risk stratification according to the COMPERA 2.0 score.

|  | Points assigned | | | |
| --- | --- | --- | --- | --- |
|  | 1 | 2 | 3 | 4 |
| WHO-FC | I or II |  | III | IV |
| 6MWD, m | >440 | 440 – 320 | 319 – 165 | <165 |
| BNP, ng/L | <50 | 50 – 199 | 200 – 800 | >800 |
| NTproBNP ng/L | <300 | 300 – 649 | 650 – 1100 | >1100 |
| Total score | = mean score (rounded to the next integer) | | | |
|  | 1 | 2 | 3 | 4 |
| Risk category | Low | Intermediate-low | Intermediate-high | High |

*WHO-FC*, World Health Organization functional class; *6MWD*, six-minute walking diastance; *BNP and NTproBNP*, brain natriuretic peptide.

**Table S3**. Characteristics of the study population according to the secondary analysis criteria for a LHD phenotype.

|  | **Overall**  **(N=286)** | **no-LHD**  **(N=206)** | **LHD**  **(N=80)** | **P** |
| --- | --- | --- | --- | --- |
| Female | 198 (69) | 58 (28) | 30 (38) | 0.16 |
| Age at diagnosis, years | 58 ± 16 | 55 ± 17 | 66 ±12 | **<0.001** |
| PAH classification | | | | 0.7 |
| IPAH/HPAH/drug induced | 139 (49) | 104 (50) | 35 (44) |  |
| CTD | 91 (32) | 63 (31) | 28 (35) |  |
| PoPH | 30 (10) | 20 (10) | 10 (12) |  |
| Other | 26 (9) | 19 (9) | 7 (9) |  |
| BMI, Kg/m^2^ | 26.4 ± 6 | 25.6 ± 5.6 | 28.7 ± 6.2 | **<0.001** |
| SBP, mmHg | 124 ± 18 | 122 ± 17 | 128 ± 19 | **0.02** |
| DBP, mmHg | 76 ± 12 | 76 ± 13 | 76 ± 11 | 0.86 |
| WHO-FC |  |  |  | 0.2 |
| I | 19 (7) | 17 (8) | 2 (3) |  |
| II | 84 (30) | 57 (28) | 27 (36) |  |
| III | 142 (51) | 103 (50) | 39 (51) |  |
| IV | 36 (13) | 28 (14) | 8 (11) |  |
| HR, bpm | 82 ± 16 | 83 ± 16 | 77 ± 13 | **0.01** |
| Hypertension | 119 (42) | 64 (31) | 55 (69) | **<0.001** |
| CAD | 38 (13) | 12 (6) | 26 (33) | **<0.001** |
| Atrial fibrillation | 41 (16) | 16 (8) | 25 (37) | **<0.001** |
| VHD | 33 (12) | 20 (10) | 13 (17) | 0.17 |
| Diabetes | 56 (20) | 19 (9) | 37 (46) | **<0.001** |
| CKD | 59 (21) | 33 (16) | 26 (33) | **0.003** |
| Synus rhythm | 262 (92) | 200 (97) | 62 (78) | **<0.001** |
| DLCO, % | 50 ± 20 | 50 ± 20 | 49 ± 20 | 0.77 |
| 6MWD, m | 308 [192 – 408] | 312 [198 – 420] | 274 [192 – 390] | 0.13 |
| Haemoglobin, g/dL | 13.9 ± 2.1 | 14.2 ± 2.1 | 13.2 ± 1.9 | **<0.001** |
| Creatinine, mg/dL | 1.04 ± 0.54 | 0.99 ± 0.43 | 1.18 ± 0.73 | **0.002** |
| NTproBNP, ng/L | 948 [261 – 2534] | 948 [248 – 2602] | 1082 [350 – 2463] | 0.78 |
| BNP, ng/L | 253 [87 – 624] | 308 [92 – 704] | 174 [72 – 511] | 0.18 |
| Baseline echocardiography | | | | |
| LVEF, % | 59 ± 7 | 60 ± 7 | 59 ± 7 | 0.78 |
| Mitral E peak, m/s | 0.53 ± 0.24 | 0.51 ± 0.23 | 0.58 ± 0.27 | 0.05 |
| Mitral E/A ratio | 0.81 ± 0.44 | 0.81 ± 0.47 | 0.79 ± 0.30 | 0.46 |
| Mitral E/e’ ratio | 8.3 ± 4.5 | 7.5 ± 3.8 | 10.6 ± 5.4 | **<0.001** |
| LA area, cm^2^ | 18 ± 6 | 17 ± 5 | 22 ± 7 | **<0.001** |
| RV basal diameter, cm | 4.8 [4.3 – 5.4] | 4.8 [4.3 – 5.4] | 4.6 [4.2 – 5.4] | 0.38 |
| TAPSE, mm | 17 ± 8 | 17 ± 9 | 20 ± 5 | **<0.001** |
| FAC, % | 28 ± 11 | 26 ± 10 | 32 ± 10 | **<0.001** |
| RVSP, mmHg | 67 ± 22 | 70 ± 23 | 60 ± 18 | **0.009** |
| eRAP, mmHg | 8 ± 4 | 8 ± 4 | 7 ± 4 | 0.42 |
| TAPSE/sPAP | 0.26 ± 0.14 | 0.24 ± 0.13 | 0.32 ± 0.13 | **<0.001** |
| RVOT-AccT, ms | 76 ± 20 | 76 ± 20 | 77 ± 19 | 0.71 |
| RA area, cm^2^ | 24 ± 7 | 24 ± 7 | 24 ± 7 | 0.73 |
| Pericardial effusion | 79 (29) | 59 (30) | 20 (26) | 0.69 |
| Baseline right heart catheterization | | | | |
| sPAP, mmHg | 75 ± 21 | 78 ± 20 | 68 ± 20 | **<0.001** |
| dPAP, mmHg | 31 ± 11 | 32 ± 11 | 26 ± 9 | **<0.001** |
| mPAP, mmHg | 47 ± 13 | 49 ± 13 | 41 ± 12 | **<0.001** |
| PAWP, mmHg | 9 ± 4 | 9 ± 3 | 11 ± 5 | **<0.001** |
| RAP, mmHg | 8 ± 4 | 8 ± 5 | 8 ± 4 | 0.59 |
| CO, L/min | 4.21 ± 1.55 | 3.95 ± 1.34 | 4.88 ± 1.84 | **<0.001** |
| CI, L/min/m^2^ | 2.38 ± 0.81 | 2.28 ± 0.67 | 2.64 ± 1.04 | **0.002** |
| PVR, WU | 10.1 ± 5.57 | 11.3 ± 5.51 | 7 ± 4.48 | **<0.001** |
| Therapy after diagnosis | | | | |
| ERA | 174 (61) | 131 (64) | 43 (54) | 0.16 |
| PDE5i/GCs | 192 (67) | 144 (70) | 48 (60) | 0.14 |
| Prostanoid | 14 (5) | 14 (7) | 0 | 0.04 |
| Dual oral | 114 (40) | 91 (44) | 23 (29) | **0.02** |
| Beta-blockers | 64 (22) | 31 (15) | 33 (41) | **<0.001** |
| ACEi/ARB | 71 (25) | 42 (20) | 29 (36) | **0.008** |
| Anticoagulation | 80 (28) | 56 (27) | 24 (30) | 0.74 |
| Amiodarone | 3 (1) | 2 (1) | 1 (1) | 1 |
| Digoxin | 10 (4) | 6 (3) | 4 (5) | 0.61 |
| Therapy at first disease reassessment | | | | |
| ERA | 206 (72) | 155 (75) | 51 (64) | 0.05 |
| PDE5i/sGC | 214 (75) | 162 (79) | 52 (65) | **0.02** |
| Prostanoid | 40 (14) | 32 (16) | 8 (10) | 0.20 |
| Dual oral | 156 (55) | 124 (60) | 32 (40) | **0.002** |

*PAH,* pulmonary arterial hypertension; *IPAH*, idiopatic PAH; *HPAH*, hereditary PAH; *CTD*, connective tissue disease; *PoPH*, porto-pulmonary hypertension; *BMI,* body mass index;  *SBP,* systolic blood pressure; *DBP,* diastolic blood pressure; *WHO-FC,* World Health Organization functional class; *HR*, heart rate; *CAD,* coronary artery disease; *VHD,* valvular heart disease; *CKD,* chronic kidney disease; *DLCO,* diffusion capacity of carbon monoxide; *6MWD*, six-minute walking distance; *LVEF*, left ventricular ejection fraction; *LA*, left atrium; *RV*, right ventricle; *TAPSE,* tricuspid annular plane systolic excursion; *FAC*, fractional area change; *RVSP,* right ventricular systolic pressure; *eRAP*, estimated right atrial pressure; *RVOT-AccT*, right ventricle outflow tract acceleration time; *RA,* right atrium; *mPAP, dPAP and sPAP* for mean, diastolic and systolic pulmonary artery pressure; *PAWP,* pulmonary artery wedge pressure; *RAP,* right atrial pressure; CO, cardiac output; CI, cardiac index; *PVR,* pulmonary vascular resistance; *ERA,* endothelin receptor antagonist; *PDE5i,* phosphodiesterase type 5 inhibitor; *GCs,* guanylate cyclase stimulator; *ACEi,* angiotensin converting enzyme inhibitors*; ARB,* angiotensin receptor blockers.

**Table S4**. Characteristics of the study population, as categorized by the presence of a LHD phenotype, when limited to patients with a diagnosis of PAH from 2013 to 2021.

|  | **Overall**  **(N=265)** | **no-LHD**  **(N=212)** | **LHD**  **(N=53)** | **P** |
| --- | --- | --- | --- | --- |
| Female | 185 (70) | 152 (72) | 33 (62) | 0.20 |
| Age at diagnosis, years | 59 ± 16 | 57 ± 17 | 66 ± 14 | **<0.001** |
| PAH classification | | | | 0.50 |
| IPAH/HPAH/drug induced | 133 (50) | 111 (52) | 22 (42) |  |
| CTD | 85 (32) | 66 (31) | 19 (36) |  |
| PoPH | 28 (11) | 21 (10) | 7 (13) |  |
| Other | 19 (7) | 14 (7) | 5 (9) |  |
| BMI, Kg/m^2^ | 26.6 ± 5.8 | 25.9 ± 5.4 | 29.2 ± 6.6 | **<0.001** |
| SBP, mmHg | 124 ± 18 | 122 ± 17 | 130 ± 19 | **0.006** |
| DBP, mmHg | 76 ± 12 | 76 ± 13 | 77 ± 10 | 0.90 |
| WHO-FC |  |  |  | 0.30 |
| I | 18 (7) | 16 (8) | 2 (4) |  |
| II | 76 (29) | 56 (27) | 20 (38) |  |
| III | 133 (51) | 107 (51) | 26 (50) |  |
| IV | 34 (13) | 30 (14) | 4 (8) |  |
| HR, bpm | 82 ± 16 | 83 ± 16 | 77 ± 14 | **0.03** |
| Hypertension | 109 (41) | 75 (35) | 34 (64) | **<0.001** |
| CAD | 37 (14) | 19 (9) | 18 (34) | **<0.001** |
| Atrial fibrillation | 36 (15) | 23 (12) | 13 (28) | **0.004** |
| VHD | 32 (12) | 23 (11) | 9 (17) | 0.20 |
| Diabetes | 54 (20) | 30 (14) | 24 (45) | **<0.001** |
| CKD | 54 (20) | 40 (19) | 14 (26) | 0.20 |
| Synus rhythm | 245 (92) | 201 (95) | 44 (83) | **0.007** |
| DLCO, % | 50 ± 20 | 50 ± 20 | 48 ± 20 | 0.60 |
| 6MWD, m | 303 [192-405] | 302 [186-406] | 307 [240-404] | 0.50 |
| Haemoglobin, g/dL | 14.0 ± 2.1 | 14.2 ± 2.2 | 13.4 ± 2.0 | **0.02** |
| Creatinine, mg/dL | 1.05 ± 0.55 | 1.02 ± 0.44 | 1.18 ± 0.86 | 0.14 |
| NTproBNP, ng/L | 1025 [269-2534] | 1144 [298-2837] | 560 [240-1440] | 0.30 |
| BNP, ng/L | 253 [89-653] | 323 [96-749] | 128 [63-257] | **0.007** |
| Baseline echocardiography | | | | |
| LVEF, % | 59 ± 7 | 59 ± 7 | 60 ± 6 | 0.60 |
| Mitral E peak, m/s | 0.53 ± 0.25 | 0.51 ± 0.23 | 0.62 ± 0.29 | **0.02** |
| Mitral E/A ratio | 0.81 ± 0.44 | 0.81 ± 0.48 | 0.81 ± 0.23 | 0.08 |
| Mitral E/e’ ratio | 8.3 ± 4.5 | 7.8 ± 3.9 | 10.5 ± 5.9 | **<0.001** |
| LA area, cm^2^ | 18 ± 6 | 17 ± 5 | 21 ± 7 | **<0.001** |
| RV basal diameter, cm | 4.8 [4.3-5.4] | 4.8 [4.3-5.3] | 4.8 [4.3-5.4] | 0.90 |
| TAPSE, mm | 17 ± 8 | 17 ± 9 | 20 ± 5 | **<0.001** |
| FAC, % | 28 ± 11 | 27 ± 11 | 33 ± 10 | **<0.001** |
| RVSP, mmHg | 67 ± 22 | 69 ± 23 | 61 ± 19 | 0.07 |
| eRAP, mmHg | 8 ± 4 | 8 ± 5 | 6 ± 3 | **0.009** |
| TAPSE/sPAP | 0.26 ± 0.14 | 0.25 ± 0.14 | 0.33 ± 0.14 | **<0.001** |
| RVOT-AccT, ms | 76 ± 20 | 75 ± 20 | 78 ± 21 | 0.50 |
| RA area, cm^2^ | 24 ± 8 | 24 ± 8 | 23 ± 7 | 0.50 |
| Pericardial effusion | 72 (28) | 60 (29) | 12 (24) | 0.50 |
| Baseline right heart catheterization | | | | |
| sPAP, mmHg | 75 ± 21 | 78 ± 20 | 64 ± 20 | **<0.001** |
| dPAP, mmHg | 31 ± 11 | 32 ± 11 | 25 ± 7 | **<0.001** |
| mPAP, mmHg | 47 ± 13 | 49 ± 13 | 39 ± 11 | **<0.001** |
| PAWP, mmHg | 10 ± 4 | 9 ± 3 | 12 ± 5 | **<0.001** |
| RAP, mmHg | 8 ± 4 | 9 ± 5 | 8 ± 4 | 0.20 |
| CO, L/min | 4.19 ± 1.49 | 3.91 ± 1.21 | 5.29 ± 1.94 | **<0.001** |
| CI, L/min/m^2^ | 2.38 ± 0.82 | 2.27 ± 0.67 | 2.82 ± 1.15 | **<0.001** |
| PVR, WU | 10.2 ± 5.7 | 11.3 ± 5.5 | 6.1 ± 4.5 | **<0.001** |
| Therapy after diagnosis | | | | |
| ERA | 159 (60) | 131 (62) | 28 (53) | 0.20 |
| PDE5i/GCs | 183 (69) | 154 (73) | 29 (55) | **0.01** |
| Prostanoid | 13 (5) | 13 (6) | 0 | 0.08 |
| Dual oral | 110 (42) | 95 (45) | 15 (28) | **0.03** |
| Beta-blockers | 60 (23) | 35 (17) | 25 (47) | **<0.001** |
| ACEi/ARB | 66 (25) | 44 (21) | 22 (42) | **0.002** |
| Anticoagulation | 69 (26) | 54 (25) | 15 (28) | 0.70 |
| Amiodarone | 3 (1) | 3 (1) | 0 | 0.90 |
| Digoxin | 7 (3) | 7 (3) | 0 | 0.40 |
| Therapy at first disease reassessment | | | | |
| ERA | 193 (73) | 162 (76) | 31 (58) | **0.009** |
| PDE5i/sGC | 202 (76) | 169 (80) | 33 (62) | **0.008** |
| Prostanoid | 37 (14) | 34 (16) | 3 (6) | 0.05 |
| Dual oral | 150 (57) | 131 (62) | 19 (36) | **<0.001** |

*PAH,* pulmonary arterial hypertension; *IPAH*, idiopatic PAH; *HPAH*, hereditary PAH; *CTD*, connective tissue disease; *PoPH*, porto-pulmonary hypertension; *BMI,* body mass index;  *SBP,* systolic blood pressure; *DBP,* diastolic blood pressure; *WHO-FC,* World Health Organization functional class; *HR*, heart rate; *CAD,* coronary artery disease; *VHD,* valvular heart disease; *CKD,* chronic kidney disease; *DLCO,* diffusion capacity of carbon monoxide; *6MWD*, six-minute walking distance; *LVEF*, left ventricular ejection fraction; *LA*, left atrium; *RV*, right ventricle; *TAPSE,* tricuspid annular plane systolic excursion; *FAC*, fractional area change; *RVSP,* right ventricular systolic pressure; *eRAP*, estimated right atrial pressure; *RVOT-AccT*, right ventricle outflow tract acceleration time; *RA,* right atrium; *mPAP, dPAP and sPAP* for mean, diastolic and systolic pulmonary artery pressure; *PAWP,* pulmonary artery wedge pressure; *RAP,* right atrial pressure; CO, cardiac output; CI, cardiac index; *PVR,* pulmonary vascular resistance; *ERA,* endothelin receptor antagonist; *PDE5i,* phosphodiesterase type 5 inhibitor; *GCs,* guanylate cyclase stimulator; *ACEi,* angiotensin converting enzyme inhibitors*; ARB,* angiotensin receptor blockers.

**Figure S1.** Survival curves in patients without (solid lines) and with (dashed lines) a LHD phenotype for each COMPERA risk stratum at baseline (a, low risk; b, intermediate risk; c, high risk).


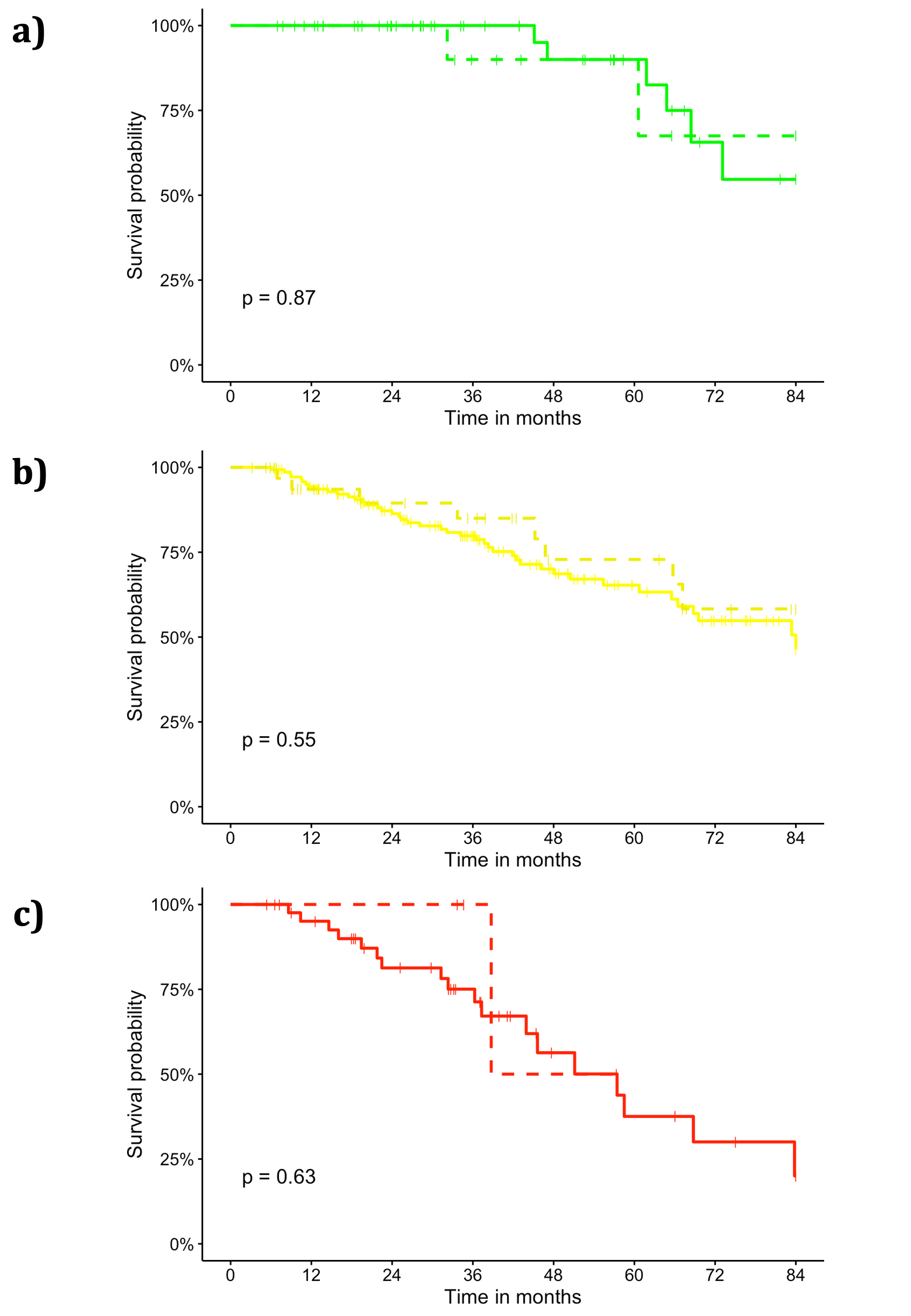


**Figure S2:** Survival curves in patients without (solid lines) and with (dashed lines) a LHD phenotype for each COMPERA risk stratum at first disease reassessment (a, low risk; b, intermediate risk; c, high risk).


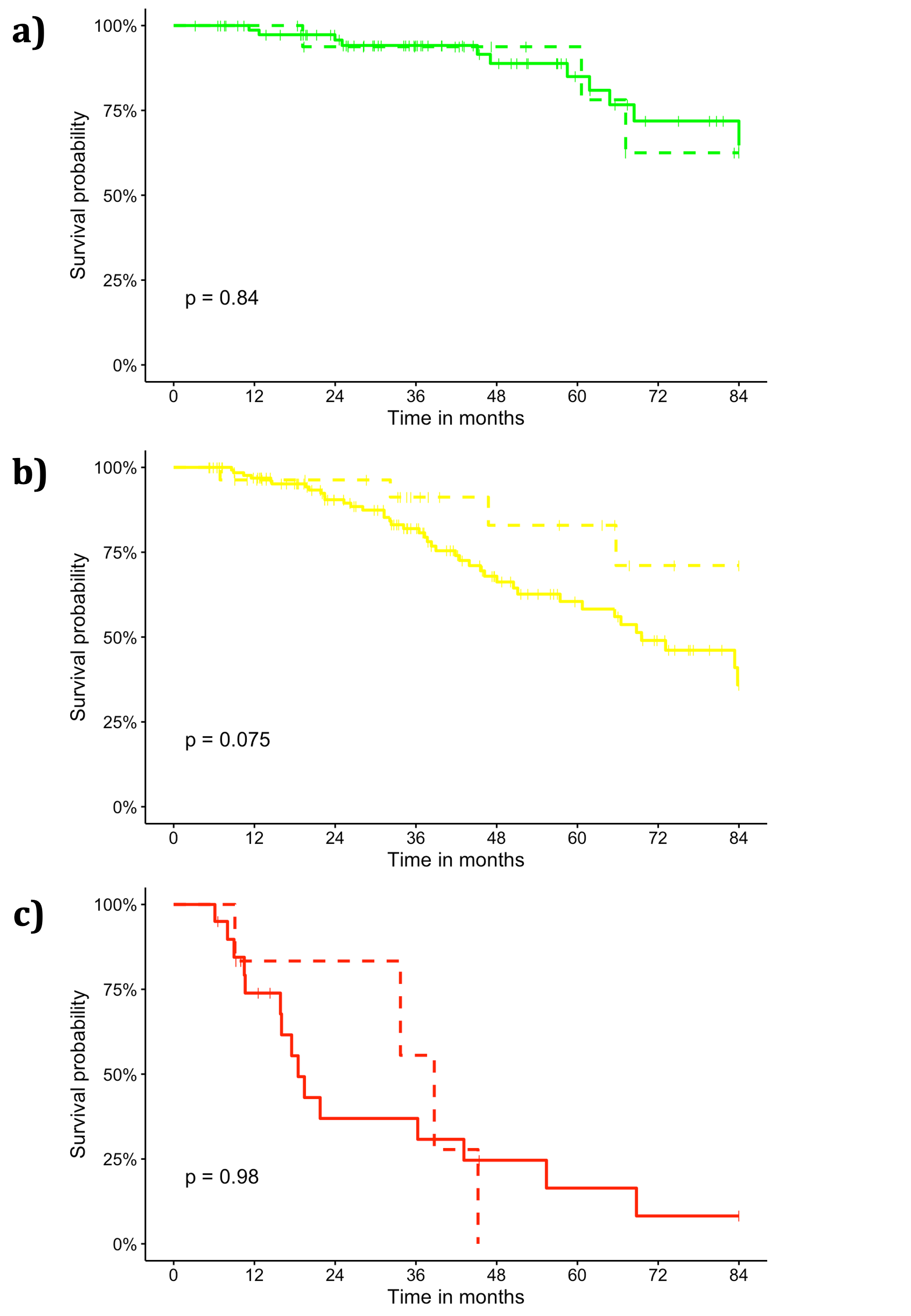


**Figure S3.** Survival curves in patients without (solid lines) and with (dashed lines) a LHD phenotype for each COMPERA 2.0 risk stratum at baseline (a, low risk; b, intermediate-low risk; c, intermediate-high risk; d, high risk).


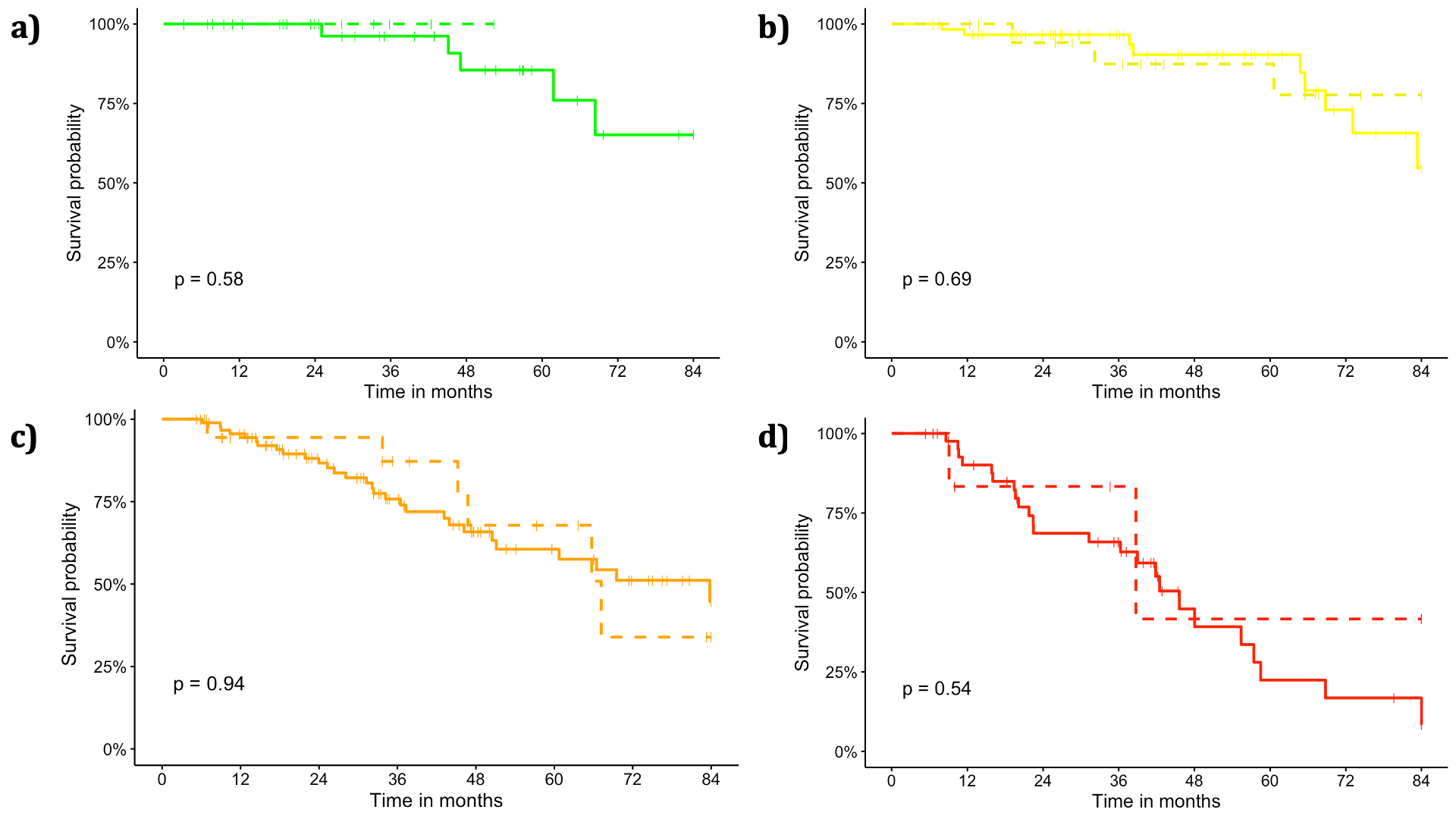


**Figure S4.** Survival curves in patients without (solid line) and with (dashed line) a LHD phenotype for each COMPERA 2.0 risk stratum at first disease reassessment (a, low risk; b, intermediate-low risk; c, intermediate-high risk; d, high risk).


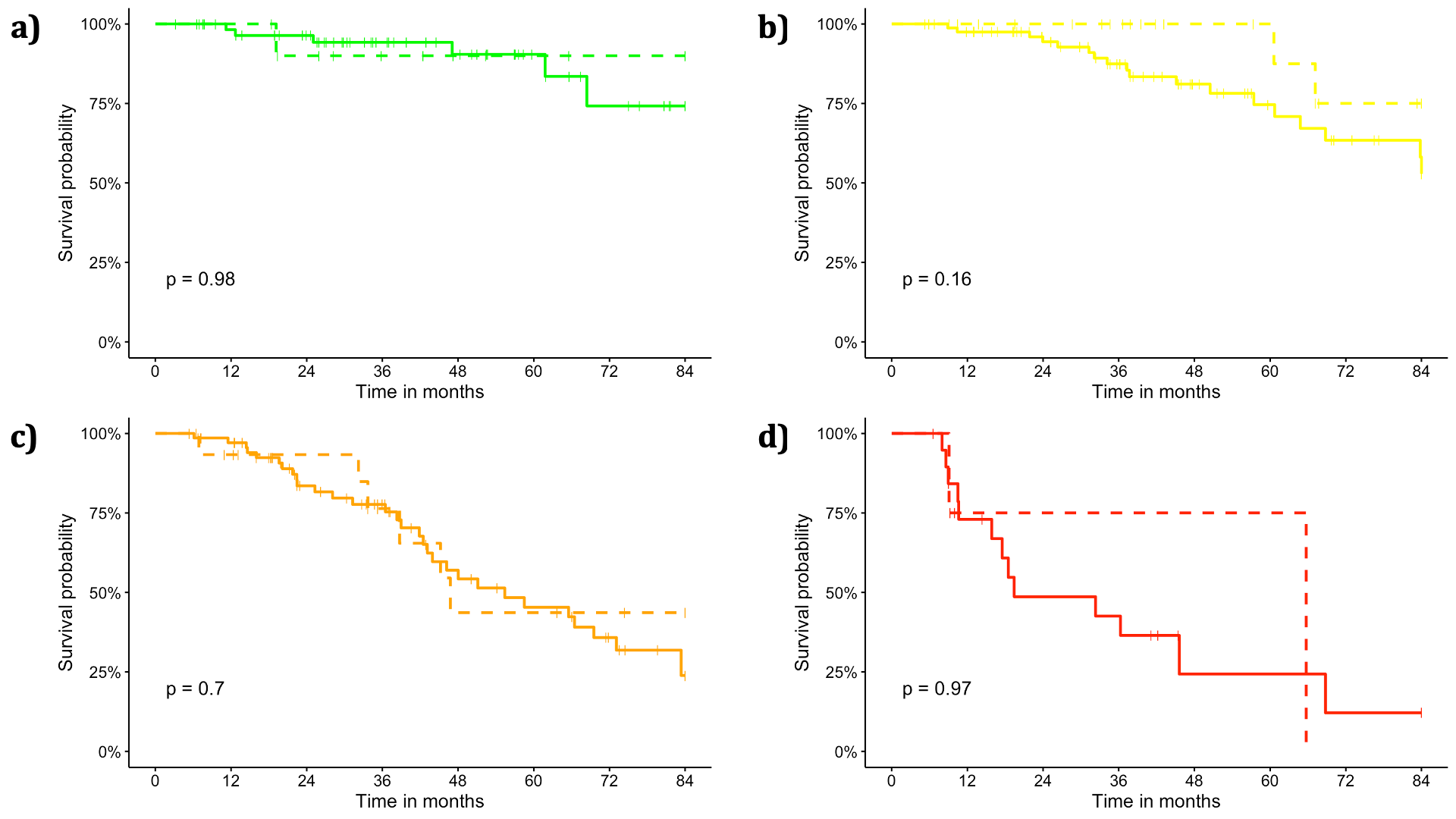


**Figure S5**. Changes in World Health Organization functional class (a), six-minute walking distance (b) and natriuretic peptide concentrations (c) in patients with (red) and without (blue) a LHD phenotype according to the secondary analysis criteria.

*** indicates P<0.001.


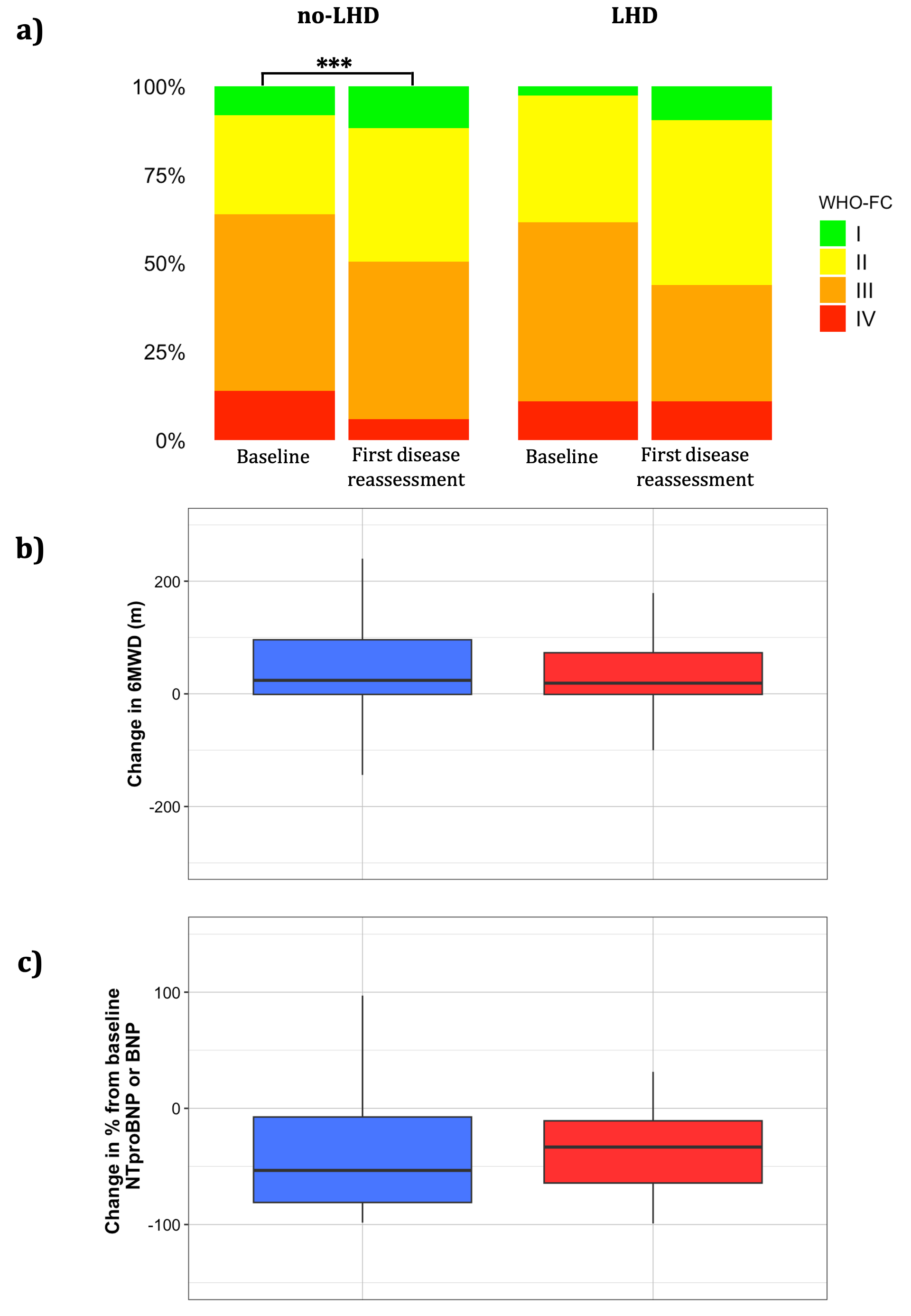


**Figure S6.** Changes in risk from baseline to first disease reassessment according to COMPERA and COMPERA 2.0 models in patients without (a) and with (b) a LHD phenotype according to the secondary analysis criteria.

*, ** and *** indicate P<0.05, P<0.01 and P<0.001, respectively

**
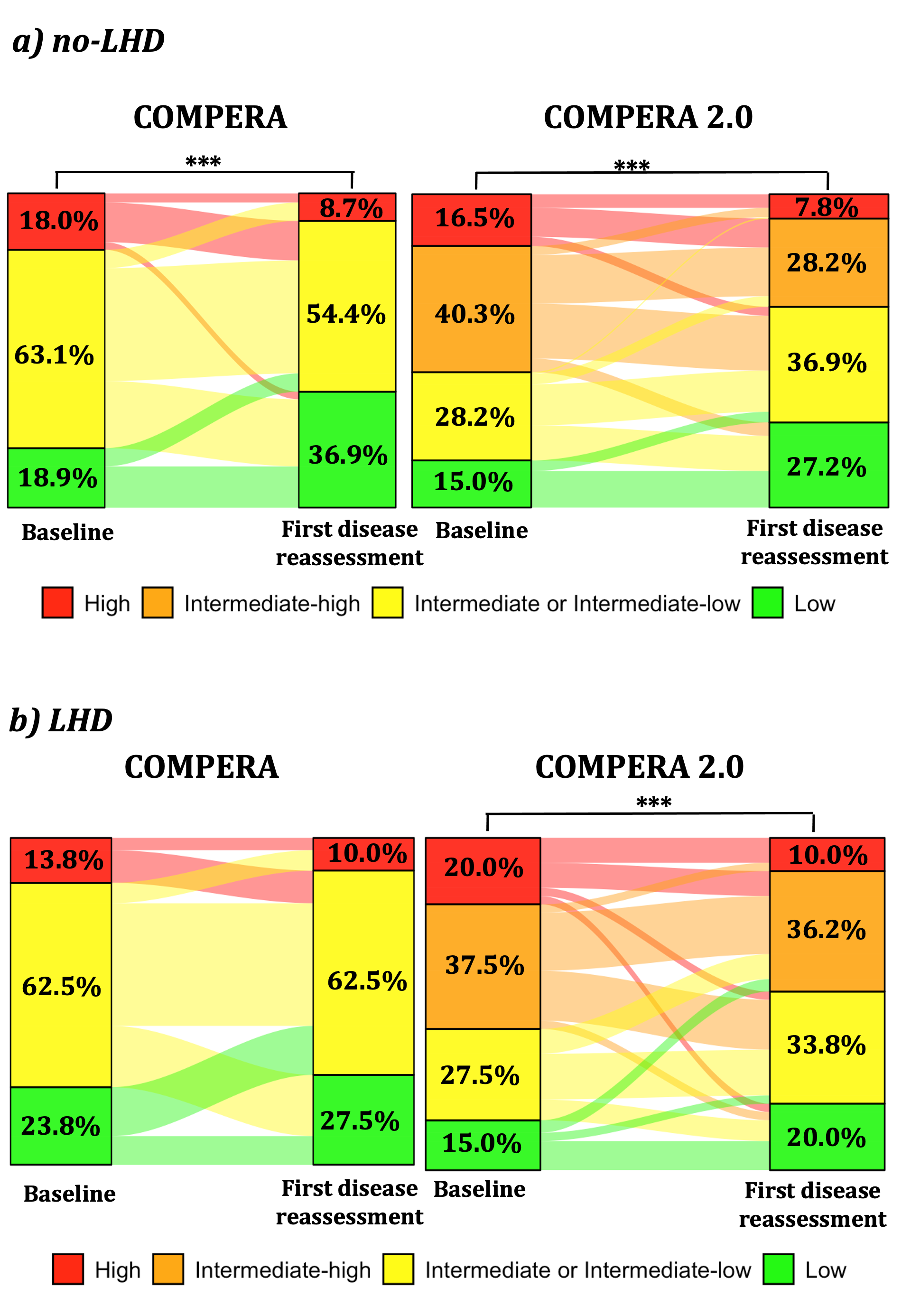
**

**Figure S7**. Survival curves according to risk strata at baseline, as assessed by baseline the COMPERA and COMPERA 2.0 models, in patients with (dashed lines) and without (solid lines) a LHD phenotype according to the secondary analysis criteria.


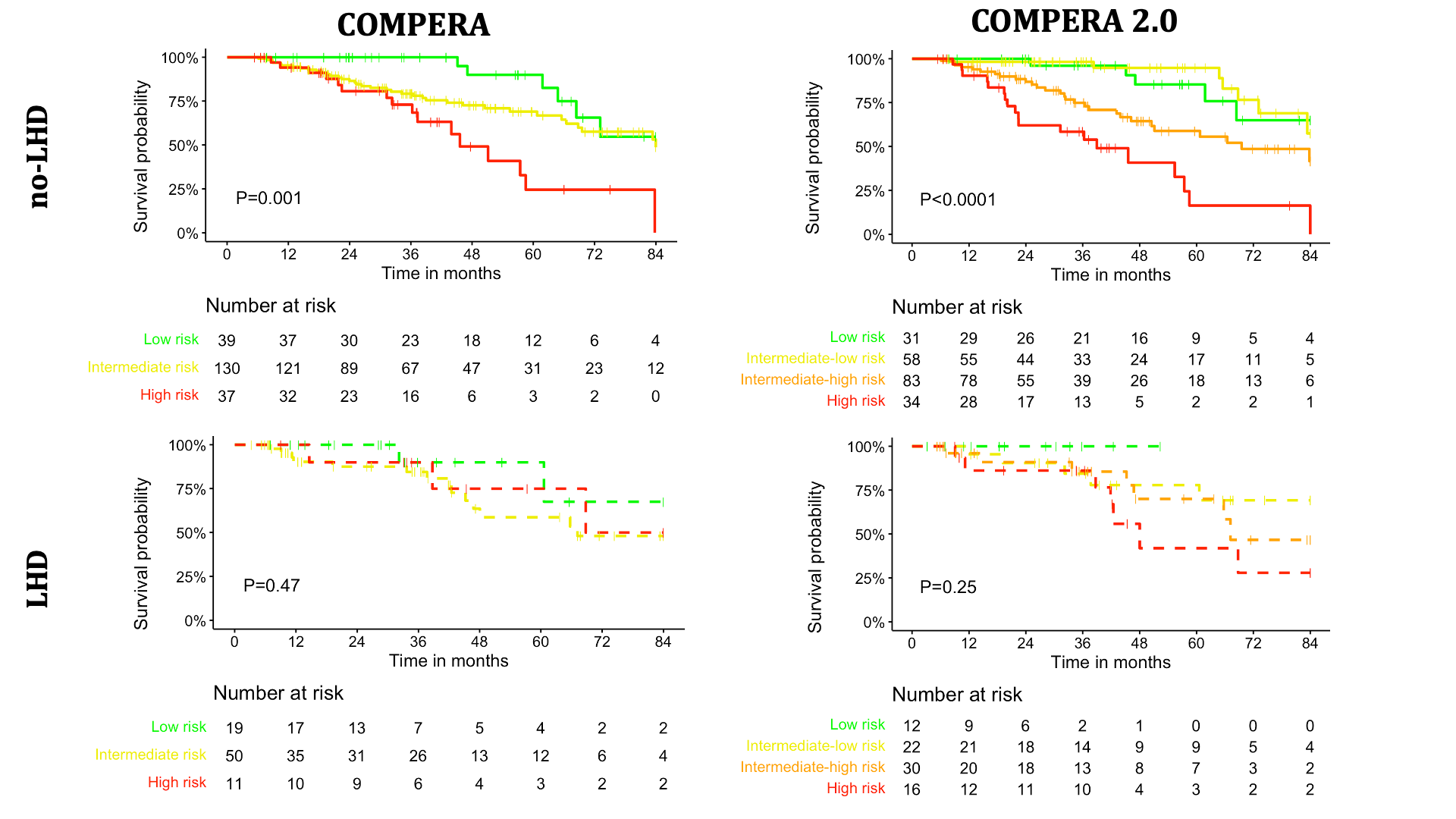


**Figure S8**. Survival curves according to risk strata at first disease reassessment, as assessed by the COMPERA and COMPERA 2.0 models, in patients with (dashed lines) and without (solid lines) a LHD phenotype according to the secondary analysis criteria.


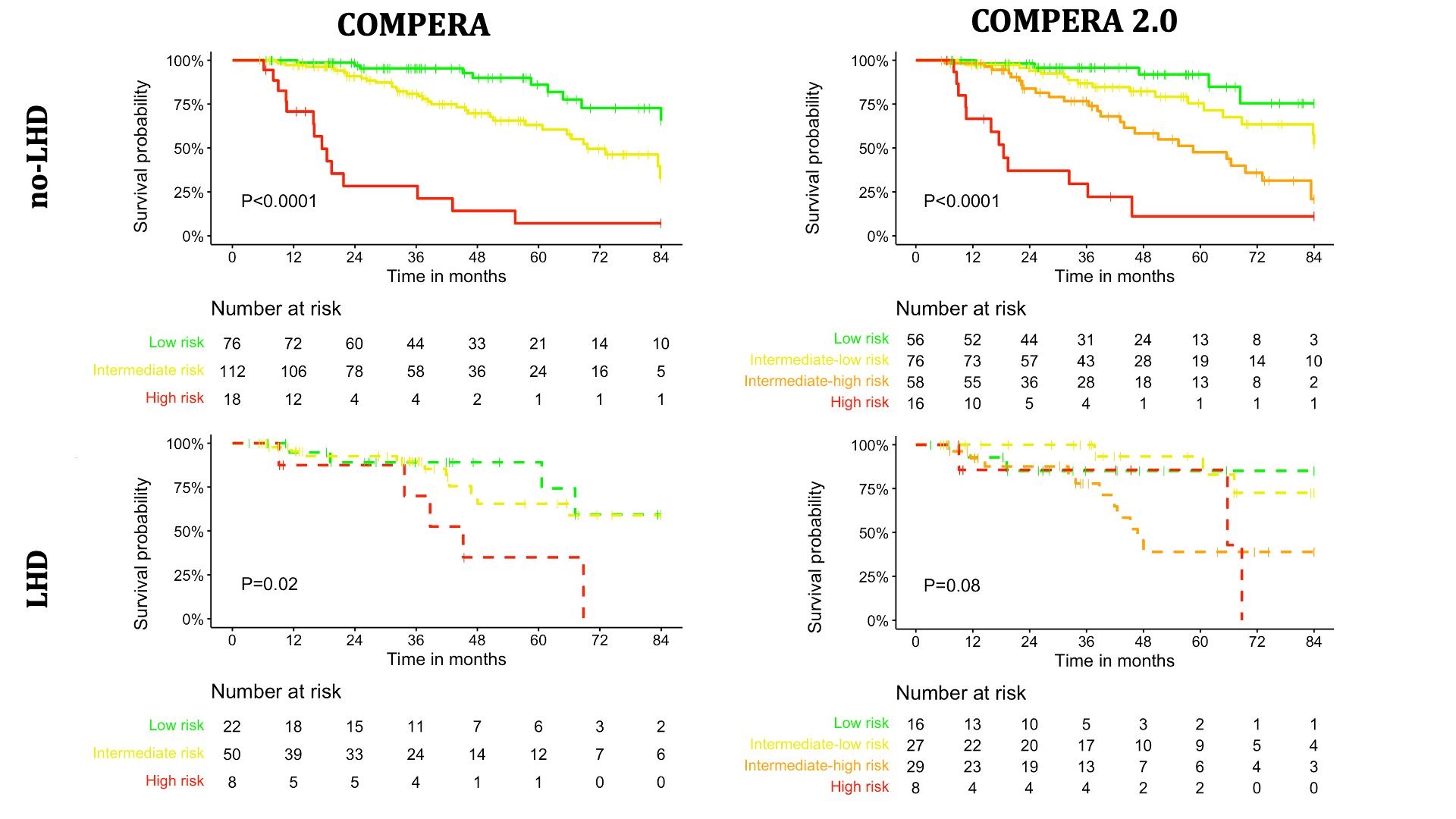


**Figure S9**. Survival curves according to risk strata at baseline, as assessed by baseline the COMPERA and COMPERA 2.0 models, in patients diagnosed between 2013 and 2021 with (dashed lines) and without (solid lines) a LHD.


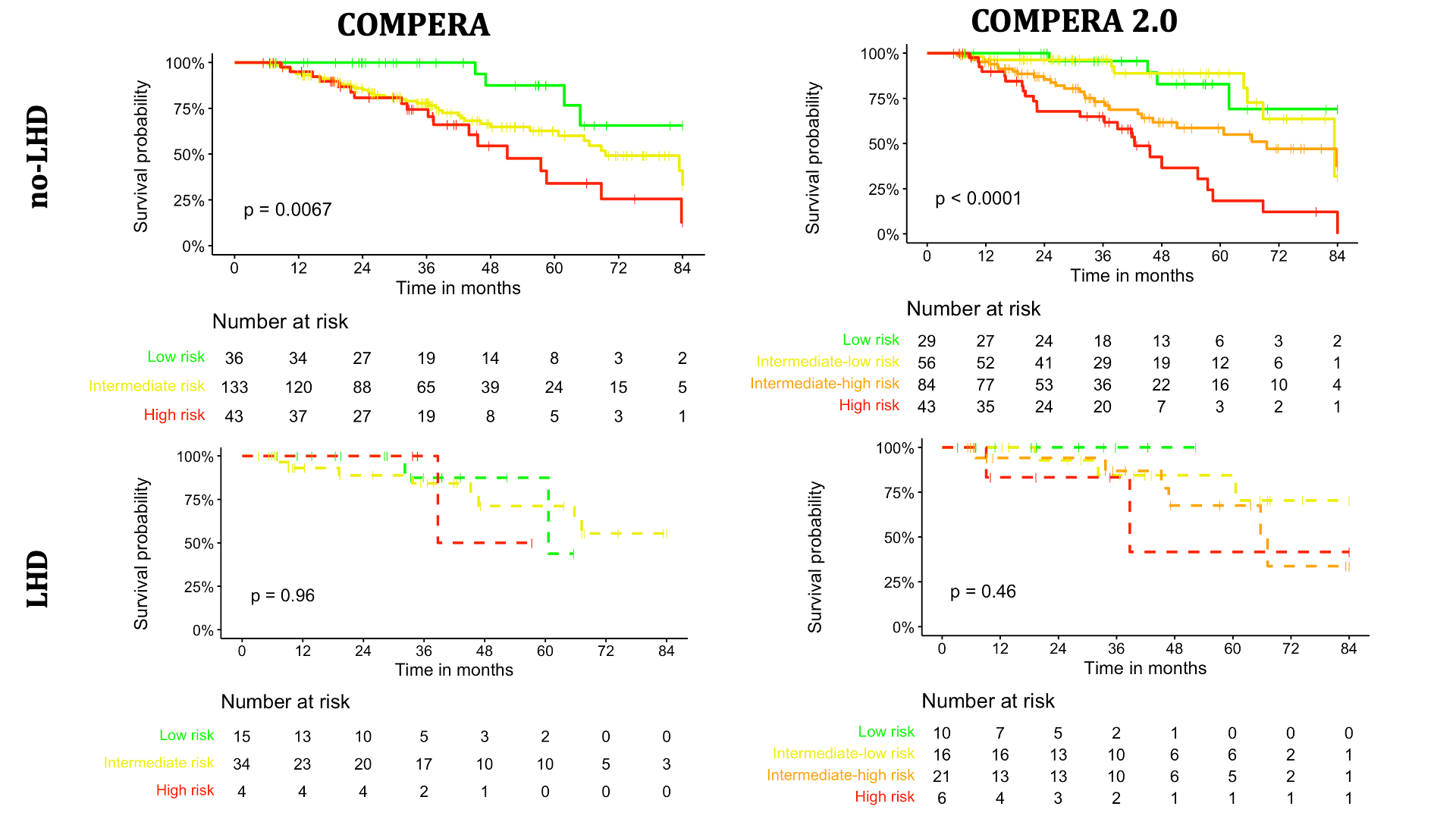


**Figure S10**. Survival curves according to risk strata at first disease reassessment, as assessed by the COMPERA and COMPERA 2.0 models, in patients diagnosed between 2013 and 2021 with (dashed lines) and without (solid lines) a LHD phenotype.


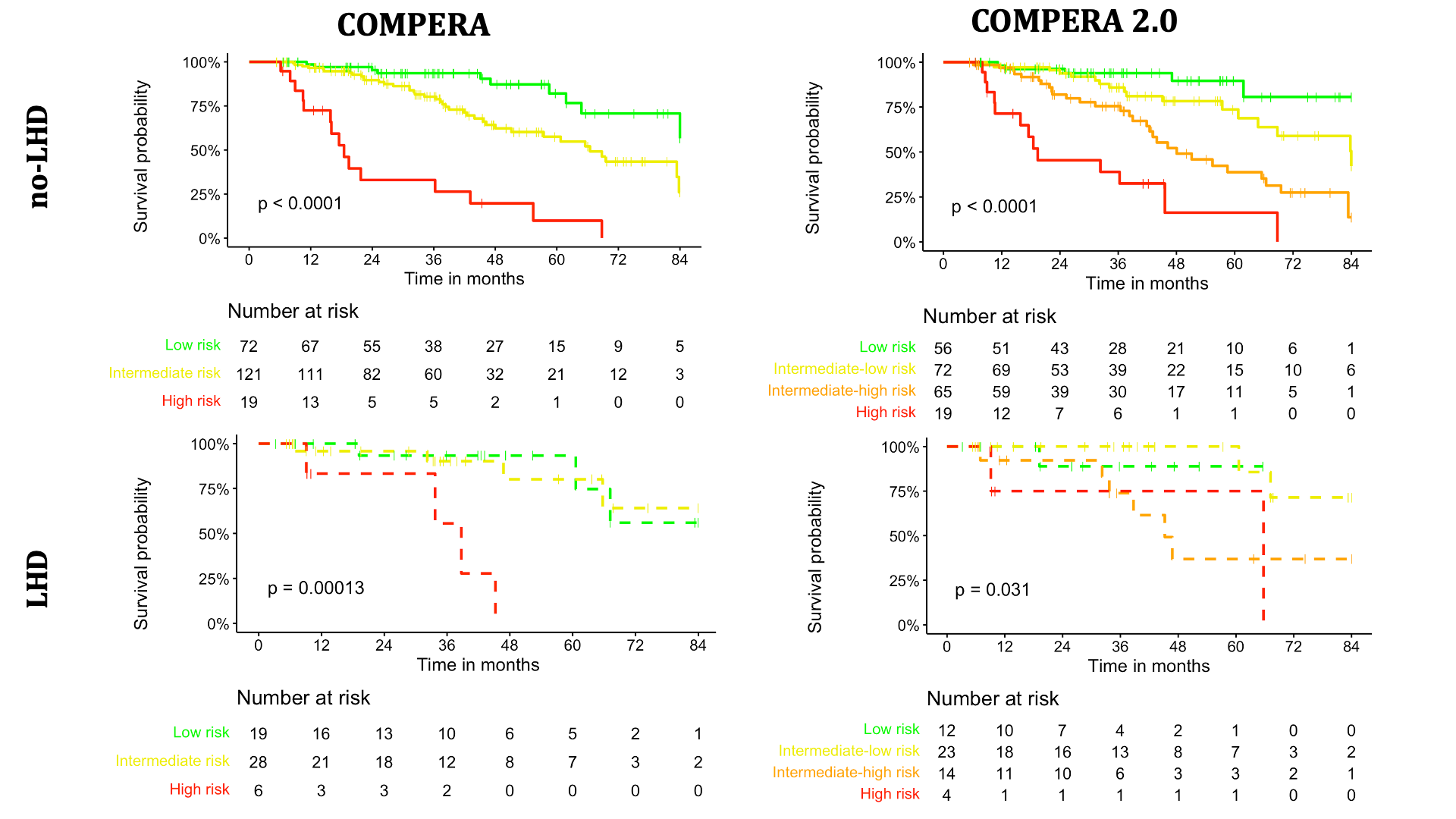

Supplement: Supplementary file 1 — Supplementary file1 (DOC 2.91 MB) [file 392_2024_2448_MOESM1_ESM.doc]
